# Effect of Frequency Response Manipulations on Musical Sound Quality for Cochlear Implant Users

**DOI:** 10.1177/23312165221120017

**Published:** 2022-08-19

**Authors:** Jonathan Mo, Nicole T. Jiam, Mickael L.D. Deroche, Patpong Jiradejvong, Charles J. Limb

**Affiliations:** 1 Davis School of Medicine, 8785 University of California, Sacramento, CA, USA; 2 Department of Otolaryngology-Head and Neck Surgery, San Francisco School of Medicine, University of California, San Francisco, CA, USA; 3 Department of Psychology, 5618 Concordia University, Montreal, QC, Canada; * These authors contributed equally to this work.

**Keywords:** cochlear implant, frequency response manipulation, music, sound quality

## Abstract

Cochlear implant (CI) users commonly report degraded musical sound quality. To improve CI-mediated music perception and enjoyment, we must understand factors that affect sound quality. In the present study, we utilize frequency response manipulation (FRM), a process that adjusts the energies of frequency bands within an audio signal, to determine its impact on CI-user sound quality assessments of musical stimuli. Thirty-three adult CI users completed an online study and listened to FRM-altered clips derived from the top songs in Billboard magazine. Participants assessed sound quality using the MUltiple Stimulus with Hidden Reference and Anchor for CI users (CI-MUSHRA) rating scale. FRM affected sound quality ratings (SQR). Specifically, increasing the gain for low and mid-range frequencies led to higher quality ratings than reducing them. In contrast, manipulating the gain for high frequencies (those above 2 kHz) had no impact. Participants with musical training were more sensitive to FRM than non-musically trained participants and demonstrated preference for gain increases over reductions. These findings suggest that, even among CI users, past musical training provides listeners with subtleties in musical appraisal, even though their hearing is now mediated electrically and bears little resemblance to their musical experience prior to implantation. Increased gain below 2 kHz may lead to higher sound quality than for equivalent reductions, perhaps because it offers greater access to lyrics in songs or because it provides more salient beat sensations.

## Introduction

Cochlear implants (CI) are surgically implanted devices that restore partial sound and speech perception to individuals with severe-to-profound sensorineural hearing loss (Carlson, 2020; Gaylor et al., 2013). While these devices have been shown to significantly improve speech perception in quiet environments (Wilson & Dorman, 2008), the processing of complex sounds such as music is severely limited (Limb, 2006). Sound quality is significantly diminished for CI users compared to normal-hearing listeners (Lassaletta et al., 2008a, 2008b; Roy et al., 2012a). Furthermore, pitch discrimination, which is important for timbre and melody recognition, is relatively poor (Gfeller et al., 2007; Looi et al., 2008; Sucher & McDermott, 2007). These perceptual challenges largely stem from technological and anatomical limitations with CIs, including reduced frequency resolution due to broad bandpass filtering, a limited CI frequency input range, and imprecise electrical stimulation of the auditory nerve (Limb & Roy, 2014). These factors may contribute to the decreased music enjoyment that many CI users experience, especially for individuals who used to enjoy music with normal acoustic hearing (Gfeller et al., 2000; Migirov et al., 2009).

Beyond the fidelity, quality, and structural characteristics of a musical signal, a myriad of other intrinsic factors play a role in influencing CI users’ enjoyment and appraisal of music. Hargreaves and colleagues described a ‘reciprocal feedback’ response model for music listening in which a complex interplay between music, the listener, and the listener's context determine emotional, cognitive, and physiological responses (Hargreaves et al., 2005). These responses are critical for forming musical likes and dislikes across short and long time spans (Hargreaves et al., 2002). The model draws from theories of aesthetic preference, including those dependent on how prototypical or familiar a stimulus is to the listener's own internal representations (Martindale et al., 1988), as well as theories of arousal state-goals for regulating emotions and achieving certain moods (North & Hargreaves, 2000). Furthermore, past studies of CI users demonstrate that performance on music perception tests is only weakly related to appraisal ratings (Gfeller et al., 2010; Wright & Uchanski, 2012). That is, the fact that an individual can perceive certain aspects and subtleties in music does not necessarily mean they will enjoy them. These findings illustrate the complex process of musical appraisal and the multi-faceted challenge of improving musical listening experiences for CI users.

From musical training paradigms to improvements in CI signal processing, a wide variety of techniques are being researched to improve both CI users’ perceptions and enjoyment of music. The present study attempts to evaluate how front-end frequency response manipulations (FRM) alter musical sound quality for CI users. FRM involves altering the relative energies of specific frequency bands within an audio signal. It is predominantly used in the audio engineering industry to improve sound quality for practical or aesthetic reasons. FRM can be used to reduce unwanted sounds or to emphasize specific instruments or voices and may improve CI users’ listening experiences.

Several studies have explored CI user musical sound quality in the past. Looi et al. (2007) compared appraisal scores for CI users and hearing-aid (HA) users in terms of the “pleasantness” of real-world musical stimuli with varying degrees of instrumental complexity (i.e., solo instruments, solo with accompaniment, small and large ensemble). CI users gave ratings that were generally higher than those of HA users and less complex (single instrument) stimuli were largely preferred. Looi et al. (2011) examined differences in musical sound quality for two CI signal processing strategies: fine-structure (FS) versus high-definition continuous interleaved sampling (HDCIS). Sound quality ratings were determined by a combination of visual analog and mid-point scales asking users to make sound quality judgements based on bipolar adjectives (e.g., unpleasant-pleasant, tinny-rich, emptier-fuller, duller-sharper). Participants demonstrated preference for the FS processing strategy over HDCIS after acclimatization to the FS strategy only. When acclimatized to the HDCIS strategy, participants showed no preference. These results suggest that using an FS processing strategy may be more beneficial for CI users’ music appreciation.

While these studies examined CI users’ musical sound quality using real-world musical stimuli, they did not directly determine sound quality differences relative to a reference signal. In order to assess sound quality differences as a result of FRM, we used the Multiple Stimulus with Hidden Reference and Anchor (MUSHRA) for CI users (CI-MUSHRA). The CI-MUSHRA is a paradigm that can systematically and quantitatively assess musical sound quality for various acoustic manipulations (Roy et al., 2012a). Participants are presented with multiple, distinct auditory stimuli derived from an unaltered reference clip. Participants then rank each clip on a scale from “0” (very poor) to “100” (excellent), based on sound quality compared to the reference. This tool reduces subjective variability because CI users evaluate differences in sound quality between clips heard within a short time, rather than rating a clip's inherent likeability or giving ratings across different testing blocks (Roy et al., 2012a). Thus far, the CI-MUSHRA paradigm has been utilized for evaluating sound quality for CI signal processing strategies (Munjal et al., 2015), insertion depth (Roy et al., 2016), reverberation (Roy et al., 2015), bass and high-frequency perception (Roy et al., 2012a; Roy et al., 2012b), and amplitude compression (Gilbert et al., 2021). The present study employed the CI-MUSHRA paradigm to evaluate how FRM alters musical sound quality for CI users.

Acoustic signals already undergo processing by CIs, many with the aim of optimizing speech comprehension. This processing is often not satisfactory for the perception of music, and thus has ramifications for CI-mediated musical sound quality ratings. CI bandpass filtering reduces the input signal's frequency range to approximately 200–8,500 Hz (Limb & Roy, 2014). The absence of frequency information outside of this range impairs musical sound quality (Moore & Tan, 2003) because music contains significant spectral energy outside that range (Roy et al., 2012a, 2012b). Moreover, frequency resolution is diminished due to current spreading and because the limited number of CI electrodes in an array cannot mimic the fine-grain tonotopy of the human cochlea (Limb & Roy, 2014). Conventional CI signal processing strategies also extract and prioritize temporal envelope information over temporal fine structure (TFS) cues, which may be important for music perception (Moon & Hong, 2014). For this reason, novel processing and electro-acoustic stimulation strategies for music listening have been developed to try to deliver more TFS cues (Moon & Hong, 2014; Sucher & McDermott, 2007).

A reduced dynamic range further contributes to the degradation of CI-mediated musical sound quality. CI users have a dramatically reduced dynamic range of approximately 6 to 30 dB compared with the 120 dB range of normal hearing individuals, in part because loudness increases exponentially as a function of electric current in electrically mediated hearing (Limb & Roy, 2014; Zeng, 2004). The granularity within this dynamic range is also degraded: CI users can only discriminate about 20 steps in level whereas normal hearing individuals can discriminate as many as 200 (Shannon, 1983; Zeng, 2004). This issue is largely physiological in nature but there are additional technological issues because the input dynamic range, defined as the range of an acoustic signal that is mapped between a CI user's detection threshold and maximum stimulation levels, is somewhat malleable as a function of the user and this leads to further compression of audio. Because music spans a much wider dynamic range and contains greater dynamic variations compared to speech, a reduced dynamic range can diminish musical sound quality for CI users (Drennan & Rubinstein, 2008; Gilbert et al., 2021; Limb & Roy, 2014). This reduction also leads to disruptions in speech and timbre perception due to the distortion of spectral shape (Drennan & Rubinstein, 2008).

A variety of front-end processing features, such as automatic sensitivity control (ASC) and adaptive dynamic range optimization (ADRO), process an acoustic signal to be both comfortable and audible for CI users (Mauger et al., 2014; Patrick et al., 2006). These features have been shown to significantly improve speech comprehension and sound quality (Mauger et al., 2014). Environmental analysis technologies have also been developed to automatically select an appropriate front-end processing feature based on CI users’ listening environments, often including a music listening program (Mauger et al., 2014). While device-level modifications to improve live music listening are currently being explored, we aimed to study whether direct manipulations to music (e.g., that could be applied on pre-recorded music) could alter sound quality. These modifications may indicate important factors for CI-mediated musical sound quality and may demonstrate a novel way of improving music listening experiences as an adjunct to the variety of live technologies currently used by CI recipients.

We hypothesized that CI-mediated musical sound quality could be improved with FRM that amplified low and mid-range frequencies. The rationale for expecting benefits from amplifying low frequencies is that they tend to be poorly represented in CIs (Limb & Roy, 2014) and music is commonly perceived as lacking bass (Jiam et al., 2017). This may be due to a variety of factors including a narrowed frequency range (Limb & Roy, 2014) and because implanted arrays do not typically reach the most apical portions of the cochlea where low frequency information is transmitted (Ketten et al., 1998). Additional intra- and postoperative implantation factors, such as suboptimal placement of the electrode array (e.g., interscalar excursions, bending and kinking of the electrode array, extracochlear electrodes), insertion depth variability, and anatomic abnormalities (e.g., cochlear malformations or ossification) may further contribute to a lack of low-frequency stimulation (Cosetti & Waltzman, 2012). A music mixing study involving CI users showed that they prefer bass and drum to be amplified relative to other instruments (Buyens et al., 2014), suggesting that CI users desire greater low-frequency gain. The rationale for expecting benefits from more intense mid frequencies is that CI users prefer vocals in musical stimuli (Buyens et al., 2014) and find music easier to follow when lyrics are present (Gfeller, 2009; Jiam et al., 2017). For music with lyrics, vocal information is often essential for understanding the story and themes of a song, making it an inextricable aspect of the music. As vocal components of music are often contained within mid-range frequencies (defined as 501–2,000 Hz in the present study), FRM that boost this frequency range might help to augment CI-mediated musical sound quality due to the increased clarity and intelligibility of speech information (Limb et al., 2022).

We further hypothesized that CI users with musical training (pre- and/or post-implantation) would be more sensitive to FRM than users without training. At least for normal-hearing individuals, musicians demonstrate improved discrimination of spectrally complex signals compared to non-musicians (Brown et al., 2017). Regression analyses have also identified pre-implantation musical training as a factor associated with perceptual ability on tests of auditory and musical perception (Gfeller et al., 2008). Moreover, short-term training paradigms that aim to improve CI users’ appreciation and perception of music have demonstrated enhancements in the appraisal of real-world melodies and timbres (Looi et al., 2012), fundamental frequency discrimination (Vandali et al., 2015) and melodic contour recognition (Galvin et al., 2007), as well as real-world melody and timbre recognition (Driscoll, 2012; Hutter et al., 2015; Jiam et al., 2019). These findings suggest that CI users with prior musical training will be more sensitive to changes in sound quality produced by FRM.

Finally, as in many studies in the CI field, we were curious about the impact of our participant demographics such as music listening habits and pre- and post-lingual deafness, as these variables have been identified as relevant to CI users’ perceptions and appraisal. Gfeller et al. (2008) demonstrated that music listening experience *after implantation* was positively associated with performance on timbre and lyrical musical excerpt recognition, as well as appraisal of instrumental music. Music listening experience *before implantation* was associated with higher appraisal of lyrical music. Moreover, speech perception outcomes often differ between pre- and post-lingually deafened CI users (Boisvert et al., 2020). There may be similar effects for outcomes related to music, as pre-lingually deafened users do not have an acoustic template for what music should sound like (Moran et al., 2016) and so these individuals may be relatively more tolerant of musical distortions or inaccuracies.

## Materials and Methods

### Participant Demographics

**Figure 1.**
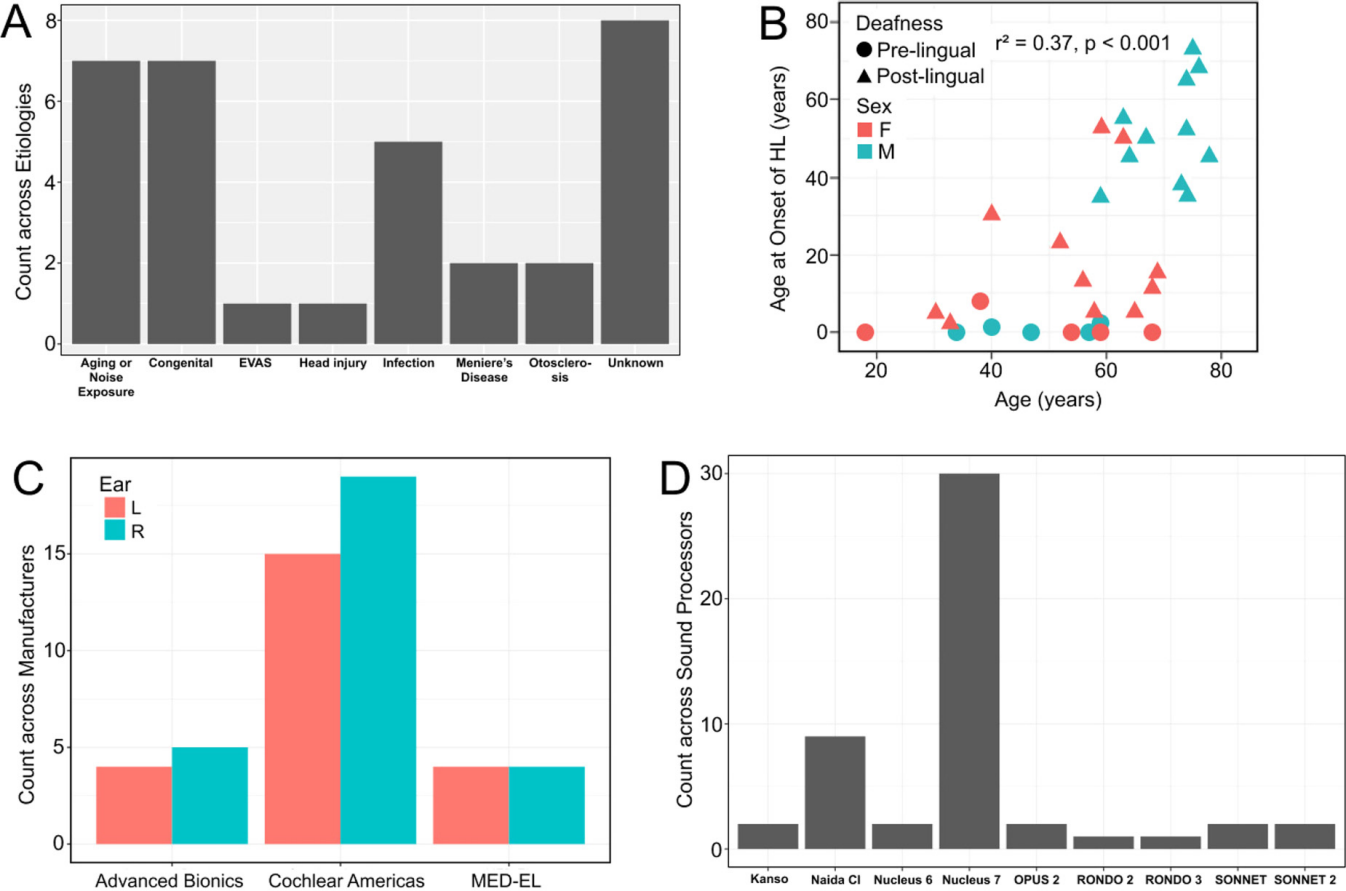
Descriptive analysis of study demographics. (A) Etiologies of hearing loss. (B) Age at onset of hearing loss as a function of age. (C) Cochlear implant manufacturer. *D,* Cochlear implant sound processor. Abbreviations: EVAS, enlarged vestibular aqueduct; F, female; HL, hearing loss; M, male.

A total of 33 CI participants completed the study. There were 16 males and 17 females. The average age was 58 ± 15 years. Participants’ etiologies of hearing loss were heterogeneous (Figure 1(A), Table 1), and the average onset of hearing loss was at 24 ± 24 years of age. The average onset of severe-to-profound hearing loss was at 38 ± 27 years of age. Ten participants were pre-lingually deafened while 23 were post-lingually deafened. Figure 1(B) shows each participant's age at onset of hearing loss as a function of age.

**Table 1.** Participant Demographics and Cochlear Implant Details.

Participant	Sex	Age (yrs)	Etiology	Ear	Age at implantation (yrs)	Implant manufacturer	Sound processor	Direct connect	Post-lingual HL	Age at onset of HL (yrs)	Age at onset of Sev-Prof HL (yrs)
1	M	34	Genetic	L	26	Cochlear	Nucleus 7	MiniMicrophone 2+	N	0	0
				R	13	Cochlear	Nucleus 7				
2	M	57	Genetic	L	41	Cochlear	Kanso	Phone Clip	N	0	0
				R	39	Cochlear	Kanso				
3	F	69	Cochlear Otosclerosis	L	50	Cochlear	Nucleus 7	iPad/iPhone	Y	15	49
				R	62	Cochlear	Nucleus 7				
4	M	64	Meniere's Disease	L	57	Advanced Bionics	Naida CI	ComPilot I	Y	40	50
				R	57	Advanced Bionics	Naida CI				
5	F	68	Idiopathic	L	58	Cochlear	Nucleus 6	Phone Clip	Y	11	54
				R	56	Cochlear	Nucleus 6				
6	F	59	Meniere's Disease	R	54	MED-EL	SONNET	AudioLink	Y	51	53
7	M	76	Aging, Noise Exposure, Genetic	L	76	MED-EL	SONNET 2	AudioLink	Y	68	74
8	F	68	Genetic	L	66	Advanced Bionics	Naida CI	CI Connect (PowerCel 170)	N	0	50
9	F	52	Idiopathic	L	46	MED-EL	RONDO 2	AudioLink	Y	23	38
				R	52	MED-EL	RONDO 3				
10	F	40	Idiopathic	R	40	MED-EL	SONNET 2	AudioLink	Y	30	33
11	M	63	Noise Exposure	L	62	MED-EL	SONNET	AudioLink	Y	55	57
12	F	33	Antibiotics	L	12	Cochlear	Nucleus 7	iPad/iPhone	Y	2	2
				R	31	Cochlear	Nucleus 7				
13	F	18	Genetic	L	1	Cochlear	Nucleus 7	MiniMicrophone 2+	N	0	0
				R	5	Cochlear	Nucleus 7				
14	F	56	Cochlear Otosclerosis	L	30	MED-EL	OPUS 2	Roger Select Transmitter + Roger MyLink	Y	13	49
				R	30	MED-EL	OPUS 2				
15	M	40	Genetic	R	40	Cochlear	Nucleus 7	Phone Clip	N	1	1
16	M	59	Aging, Noise Exposure	L	58	Cochlear	Nucleus 7	iPad/iPhone	Y	35	50
				R	58	Cochlear	Nucleus 7				
17	M	47	Genetic	L	45	Advanced Bionics	Naida CI	ComPilot I	N	0	0
				R	46	Advanced Bionics	Naida CI				
18	F	31	Infection	L	10	Cochlear	Nucleus 7	iPad/iPhone	Y	5	8
19	F	54	Genetic	L	44	Cochlear	Nucleus 7	iPad/iPhone	N	0	1
				R	45	Cochlear	Nucleus 7				
20	M	78	Aging	L	78	Cochlear	Nucleus 7	Phone Clip	Y	45	73
				R	73	Cochlear	Nucleus 7				
21	F	69	Idiopathic	L	51	Cochlear	Nucleus 7	MiniMicrophone 2+	Y	16	28
				R	59	Cochlear	Nucleus 7				
22	M	74	Aging	R	74	Advanced Bionics	Naida CI	CI Connect (w/ PowerCel 170)	Y	52	73
23	F	58	Idiopathic	L	50	Cochlear	Nucleus 7	MiniMicrophone 2+	Y	5	40
				R	50	Cochlear	Nucleus 7				
24	M	75	Head Injury	L	74	Cochlear	Nucleus 7	MiniMicrophone 2+	Y	73	73
				R	74	Cochlear	Nucleus 7				
25	F	63	Aging	R	57	Cochlear	Nucleus 7	iPad/iPhone	Y	50	52
26	M	59	Idiopathic	R	46	Cochlear	Nucleus 7	Phone Clip	N	2	2
27	M	73	Infection	R	73	Advanced Bionics	Naida CI	Roger Select Transmitter + Roger 17 Receiver	Y	38	65
28	F	59	Genetic	L	48	Advanced Bionics	Naida CI	ComPilot I	N	0	40
				R	54	Advanced Bionics	Naida CI				
29	F	65	Infection	L	64	Cochlear	Nucleus 7	MiniMicrophone 2+	Y	5	60
30	M	74	Idiopathic	R	73	Cochlear	Nucleus 7	iPad/iPhone	Y	65	72
31	M	74	Noise Exposure	R	72	Cochlear	Nucleus 7	iPad/iPhone	Y	35	35
32	F	38	Idiopathic	L	38	Cochlear	Nucleus 7	MiniMicrophone 2+	N	8	8
				R	37	Cochlear	Nucleus 7				
33	M	67	Noise Exposure, Infection	R	67	Cochlear	Nucleus 7	iPad/iPhone	Y	50	60

Abbreviations: F, female; HL, hearing loss; L, left; M, male; R, right; Sev-Prof, severe to profound.

Eighteen participants were bilateral CI users while the remaining 15 were unilateral CI users; five had left CIs and 10 had right CIs. Twenty-one participants used CIs manufactured by Cochlear, six by Advanced Bionics, and six by MED-EL. CI sound processors and direct connect equipment are described in Figure 1(C), (D), and Table 1.

### Musical Experience Demographics

**Figure 2.**
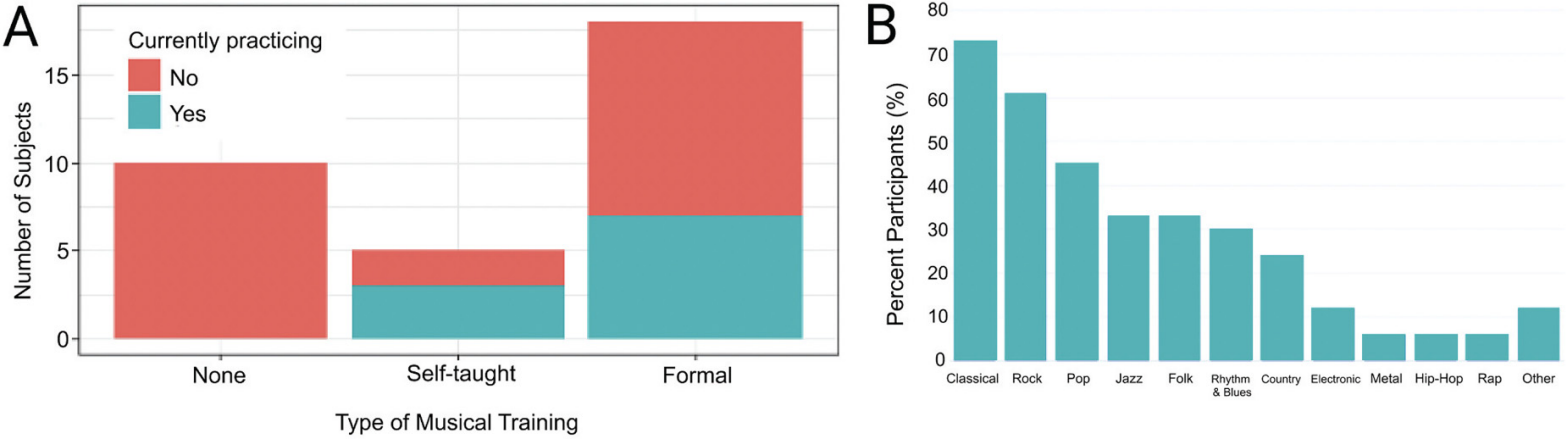
Descriptive analysis of participants’ musical experiences. (A) Type of musical training. (B) Favorite musical genres. Participants could select as many genres as they wished. Results for each genre are given as a percentage of the study population (*n* *=* *33)*.

A musical experience questionnaire was used to assess pre- and post-implantation musical training backgrounds, as well as current music listening habits. Information about initial training age, length, and type of training (i.e., no training, self-taught, or formal lessons) were recorded. The questionnaire also asked about participants’ favorite musical genres to listen to as well as their average number of hours per week of music listening. Twenty-three participants had musical training (an average training duration of 19 ± 21 years, with a minimum of two years) while 10 had no training (Figure 2(A)). Participants listened to a wide variety of musical genres with “Classical” as the most frequent favorite (Figure 2(B)) and, on average, listened to music for 10 ± 12 h per week (Table 2).

**Table 2.** Musical Experience Demographics.

Participant	Type of musical training	Instrument (Primary/Secondary)	Start age of training (yrs)	Length of training before implantation (yrs)	Length of training after implantation (yrs)	Currently playing an instrument	Average hrs/week of playing an instrument	Current hrs/week spent listening to music
1	Formal	Piano	14	0	6	Y	7	7
2	Formal	Saxophone/Violin	9	4	0	N	3	2
3	Formal	French Horn	12	7	0	N	2	2
4	Self-taught	Piano	60	0	3	N	5	8
5	None							3
6	Formal	Piano	7	47	5	Y	5	3
7	Formal	Violin/Ukulele	12	3	0	N	7	2
8	Formal	Piano	6	8	0	N	3	1
9	None							12
10	Formal	Guitar	10	8	0	Y	2	14
11	Formal	Bass/Guitar	12	30	0	Y	5	1
12	None							3
13	Formal	Piano/Violin	7	0	6	N	5	7
14	Formal	Clarinet/Saxophone	8	10	0	N	0	10
15	Self-taught	Guitar	8	10	0	N	15	30
16	Self-taught	Guitar/Bass	30	20	0	Y	5	20
17	None							6
18	None							2
19	None							18
20	Self-taught	Flute/Violin	10	2	0	Y	2	15
21	Formal	Piano	6	15	0	Y	14	1
22	Formal	Voice/Piano	8	64	0	N	6	10
23	Formal	Percussion	10	40	8	N	1	20
24	None							20
25	Formal	Piano	8	5	0	Y	6	60
26	Formal	Flute	10	7	0	N	8	20
27	Formal	Trombone/Piano	7	5	0	Y	3	2
28	Formal	Piano/Flute	6	11	0	N	15	1
29	Self-taught	Violin/Mandolin	15	49	1	Y	3	3
30	Formal	Voice/Organ	7	60	0	N	5	4
31	None							4
32	None							3
33	None							0

Abbreviations: Hrs, hours; N, no; Y, yes; Yrs, years.

Length of training before and after implantation is defined as the cumulative training time in years for both primary and secondary instruments (if applicable). Participants who currently play an instrument estimated their current average hours/week of playing. Participants who are not currently playing an instrument estimated their average hours/week of playing during training.

### Recruitment

Participant recruitment was conducted by contacting individuals who had taken part in prior CI research studies at the University of California-San Francisco and had given consent for recontact. Recruitment was also performed by posting announcements to various hearing loss and CI advocacy groups, including the Hearing Loss Association of America chapters, CI user forums and social media groups (e.g., Facebook), other CI research laboratory participant databases, and audiology clinics across the nation. The inclusion criteria were English-speaking adult (18+ years of age) CI users and the exclusion criteria were those with visual impairments, neurological conditions, or missing CI direct connect equipment.

### Musical Stimuli

Musical stimuli were derived from the ten most popular music songs in Billboard magazine taken from each five-year interval between 1970 and 2015 (Table 3). Each of the songs spent the greatest number of weeks as the top single of its year and contained both vocals and instrumentals. Original lossless, studio-quality song files were obtained from Tidal (Aspiro; Oslo, Norway; https://tidal.com/) and for each top song, a representative 7 second clip was trimmed from the song's chorus section. Each clip was pre-processed with audio software (Audacity; audacityteam.org/) to convert files from stereo to mono, to adjust the gain to prevent clipping, and to apply a 250-ms fade-in and fade-out.

**Table 3.** Song Stimuli.

Song	Title	Artist	Release year	Tempo (beats/minute)	Sex of lead vocalist	Other instruments used	Sound quality characteristics
A	Bridge Over Troubled Water	Simon & Garfunkel	1970	83	M	Drum kit, Bass, Violins	Fully acoustic, Slow moving and melodic vocal line with violin harmonies, March-like beat
B	Love Will Keep Us Together	Captain & Tennille	1975	130	F	Drum kit, Bass, Piano, Background Vocals (F)	Beat and bass-driven, Highly rhythmic, Moderate movement in vocal line with runs
C	Call Me	Blondie	1980	143	F	Drum kit, Bass, Guitar, Electric Keyboard, Background Vocals (M)	Beat and bass-driven, Highly rhythmic with simple structure, Simple and upfront vocal line
D	Like a Virgin	Madonna	1984	120	F	Drum kit, Bass, Electric Keyboard, Strings (pizzicato)	Beat and bass-driven, Highly rhythmic with simple structure, Walking bassline, Sparse background instrumentals
E	Nothing Compares 2 U	Sinéad O’Connor	1990	62	F	Drum kit, Strings	Simple rhythms, Slow moving vocal line with minimal melodic movement, Sparse background instrumentals
F	Macarena (Bayside Boys Remix)	Los Del Rio	1993	103	M	Drum kit, Bass, Electric Keyboard (with reverberation)	Beat and bass-driven, Highly rhythmic, Vocals in Spanish
G	Maria Maria	Santana ft. The Product G&B	1999	98	M	Drumkit, Bass, Acoustic Guitar	Walking bassline and bass-driven, Substantial melodic variation in vocal line with runs, Sparse background instrumentals
H	We Belong Together	Mariah Carey	2005	140	F	Drum kit, Bass, Piano, Background Vocals (F)	Beat and bass-driven, Sparse background instrumentals
I	TiK ToK	Kesha	2009	120	F	Drum kit, Bass, Electronics/Synthesizer	Beat and bass-driven, Melodic variation in vocal line, Electric-sounds/distortions of background instrumentals
J	Uptown Funk	Mark Ronson ft. Bruno Mars	2014	115	M	Drum kit, Bass, Horn Section (Trumpets, Saxophone, Low Brass), Electronics/Synthesizer, Background Vocals (M)	Beat and bass-driven, Vocals have little melodic variation and are in a spoken-style, Upfront horn harmonies, Some presence of electronics/synthesizer

Abbreviation: ft., featuring; M, male; F, female.

All songs are classified within the popular music genre.

**Figure 3.**
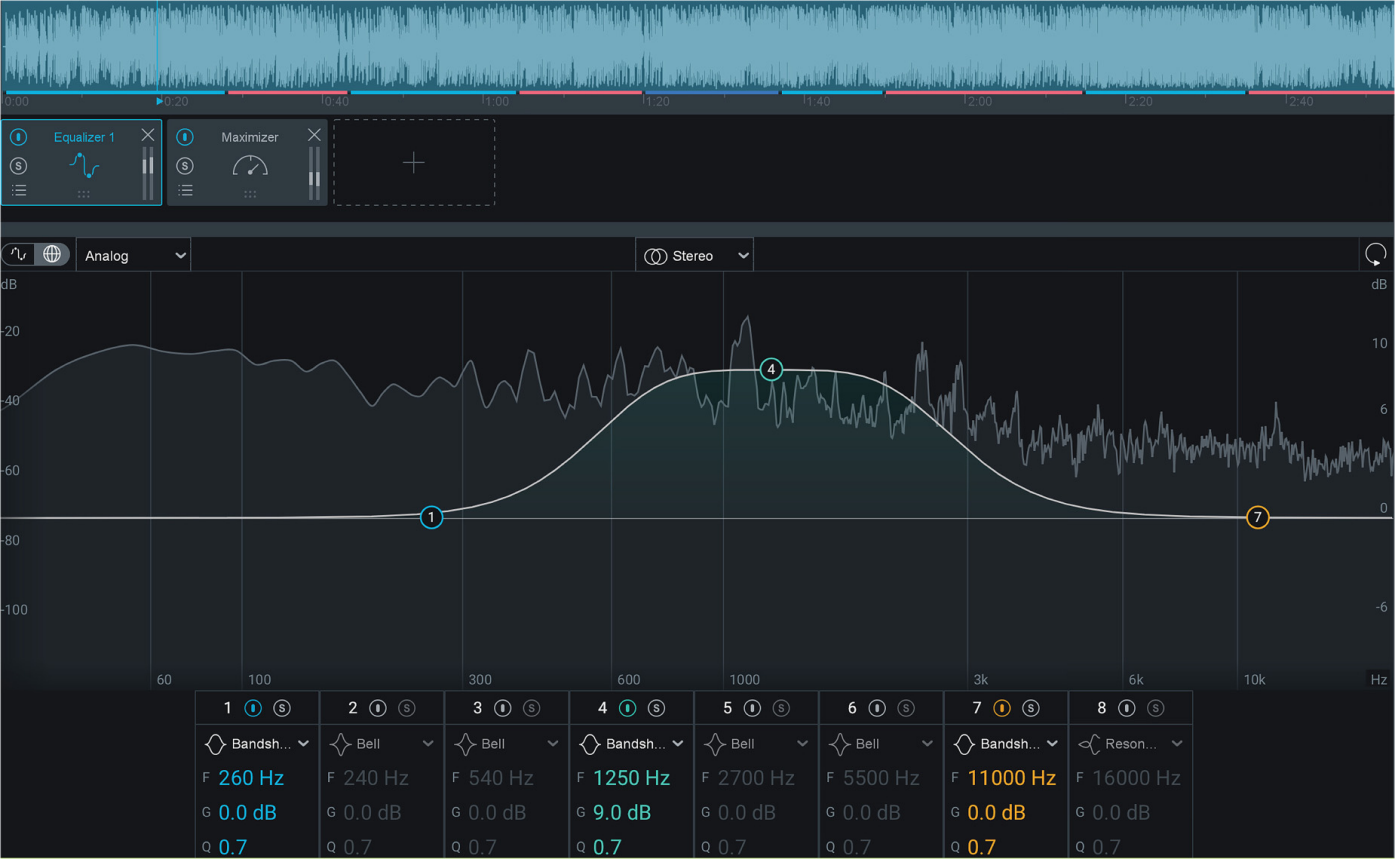
Izotope Ozone 9 interface. Gain adjustments (± 9 dB) were made in three distinct frequency ranges: Low (20–500 Hz), Medium (501–2,000 Hz), or High (2,001–20,000 Hz), resulting in six modified clips. For each manipulation, a band shelf filter (Q = 0.7) was applied at the median of a given frequency range.

FRM were performed using iZotope Ozone 9 (Cambridge, MA) audio mastering software. For each 7 second audio clip, eight versions were created. Six were made by increasing the gain (+9 dB, band shelf filter, Q = 0.7) or reducing the gain (−9 dB, band shelf filter, Q = 0.7) at the center frequency of three ranges, determined by common use within audio engineering: low (20–500 Hz), medium (501–2,000 Hz), and high (2,001–20,000 Hz). Because one goal of this study was to assess how front-end manipulations of stimuli would affect CI users’ sound quality assessments, alterations to a wide range of frequencies were chosen. These manipulations resulted in the following clips: LowDown, LowUp, MediumDown, MediumUp, HighDown, HighUp. An example interface used to create the FRM is shown in Figure 3.

Along with the six FRM for each song, corresponding reference and anchor stimuli were created. The reference stimulus consisted of the audio clip without any further adjustment. The anchor was created by applying a 1,000 Hz low-pass filter and uniformly distributed white noise to the reference with a signal-to-noise ratio of 16 dB. After these manipulations, all stimuli were root-mean-square (RMS) power normalized within each MUSHRA testing block to adjust amplitude to a standard level using Normalize (Version 0.7.7; http://normalize.nongnu.org/).

### Online Survey and Test Paradigm

This survey study was approved by an institutional review board at the University of California-San Francisco. The study protocol was distributed in an online, anonymous format and electronic informed consent was obtained from all participants. The survey, hosted on the Qualtrics XM platform (SAP; Provo, Utah), consisted of four main sections (Supplemental Material): a direct connect survey, a CI questionnaire, a musical experience questionnaire, and the CI-MUSHRA test. Participants were provided with instructions on how to set up and use direct connect equipment. Equipment-specific direct connect instructions and a testing interface video (Supplemental Material) were provided. Bilateral CI participants were asked to complete the study using their better hearing ear. Participants with electroacoustic stimulation strategies were instructed to turn off their hearing aids during testing. Participants were not required to turn off optional front-end processing features, e.g., some could have listened with their default program (including an automatic scene selection program) while others could have chosen to switch to a music program at the start of the study.

**Figure 4.**
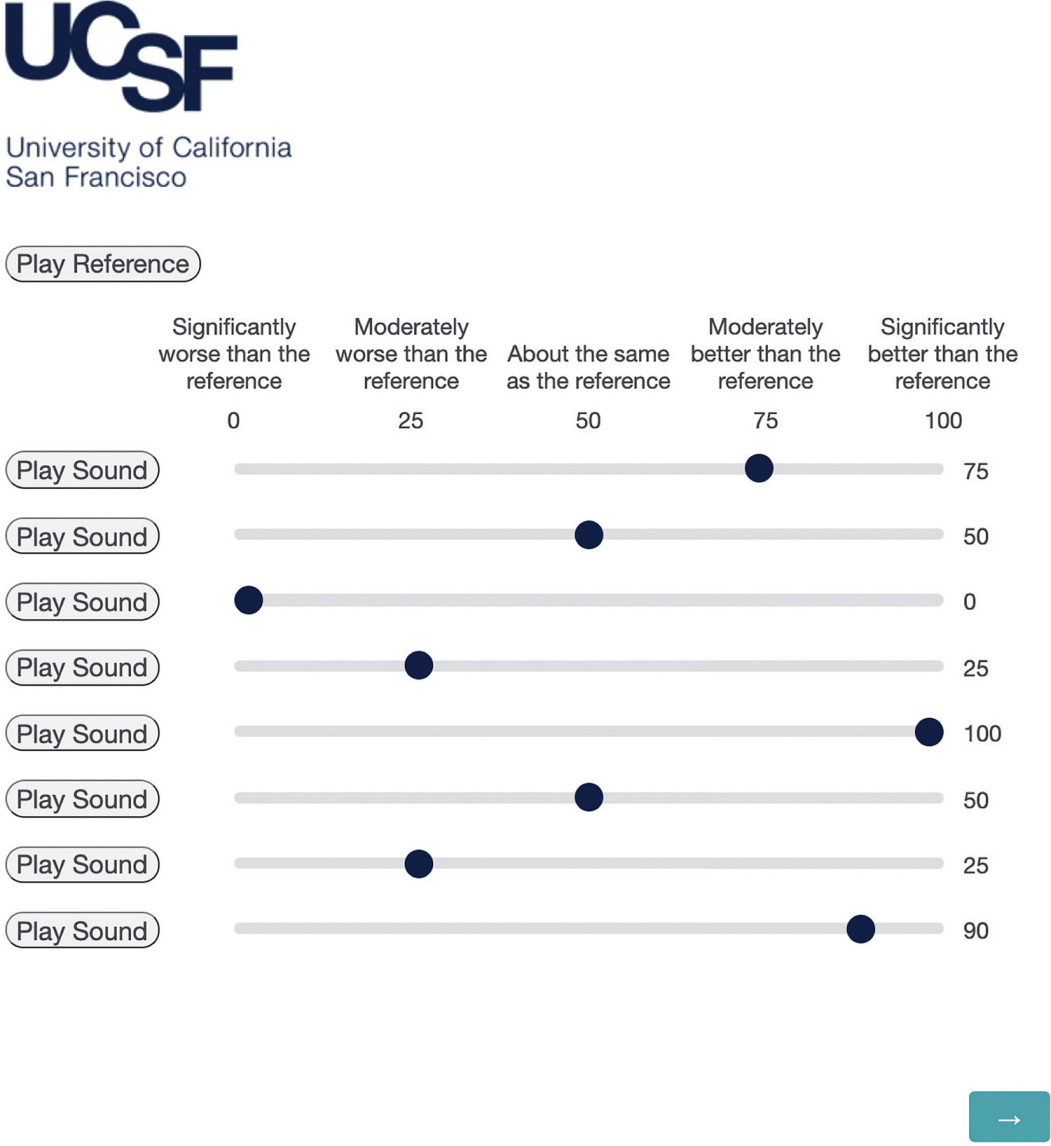
MUSHRA testing interface. Participants ranked eight audio stimuli on a sound quality rating scale from 0 to 100, comparing each to a labeled reference audio clip (top left as “Play Reference”). Each block of eight stimuli was derived from one of 10 songs and included the following, all of which were blind to participants: LowUp, LowDown, MediumUp, MediumDown, HighUp, HighDown, reference, anchor. Audio clips and song blocks were randomized for each participant. Participants were required to rank at least one clip at “0” before proceeding to the next page. Abbreviation: MUSHRA, Multiple Stimuli with Hidden Reference and Anchor.

The CI-MUSHRA paradigm was used to assess the musical sound quality ratings of stimuli with FRM relative to an unaltered reference audio clip (Figure 4). A separate testing block was used for each of the ten songs. Within each block, participants rated the perceived musical sound quality of each of the eight stimuli on a sliding scale from 0 to 100 with categorical markers (0: Significantly worse than the reference, 25: Moderately worse than the reference, 50: About the same as the reference, 75: Moderately better than the reference, 100: Significantly better than the reference). Participants did not know what stimulus was being tested. Test block and stimulus order were randomized for all participants.

The purpose of the anchor stimulus (a heavily degraded version of the reference) was to encourage a wider use of the 100-point rating scale (Roy et al., 2012a). Each testing block required participants to rank at least one clip at “0” before moving on. This baseline served as a form of interparticipant calibration and further encouraged wider use of the CI-MUSHRA rating scale.

### Statistical Analyses

Analyses were performed in R (R Core Team; Vienna, Austria) using the “dplyr” (Wickham et al., 2018) and “tidyr” (Wickham & Henry, 2020) packages for data manipulation, the “lme4” package (Bates et al., 2015) for the statistical analyses, and the “ggplot2” package (Wickham, 2016) for plotting figures. The first statistical approach used a linear mixed-effect model with sound quality rating (SQR) as the dependent variable and the eight experimental conditions as the independent variable (fixed factor). Random intercepts by participant [χ^2^(1) = 85.1, *p* < 0.001] were included but not random intercepts by item [χ^2^(1) = 2.3, *p* = 0.131]. Note that by-participant random slopes could have improved the model further but at the cost of much complexity [χ^2^(35) = 380.1, *p* < 0.001]. Instead, we opted for a simpler model and looked specifically at which participants’ characteristics moderated the effect of FRM on SQR (pre/post lingual, listening habits, and listening time). By-item random slopes never improved the model [χ^2^(35) = 25.7, *p* = 0.874] suggesting that the effect of condition was relatively homogeneous across the 10 songs (see Supplementary Material). A chi-square test compared the model without and with *condition* to assess its main effect. Post-hoc pairwise comparisons were conducted using the “emmeans” package (Lenth, 2020), with Tukey adjustments for multiple comparisons. In a second step, *musicianship* was added as a fixed factor (10 vs. 23, pooling the self-taught participants with those who received formal training). Our hypothesis was that musicianship would interact with the effect of condition. The third approach analyzed the six FRM only (i.e., the model excluded anchor and reference) in order to better elucidate the effects of the direction of the manipulation (increase vs. decrease) and the frequency range altered. This time, the model consisted of three fixed factors (*musicianship* as defined earlier, *gain* being either up or down, and *frequency range* being either low, mid, or high), preserving earlier random intercepts by participant. To analyze additional aspects of the participant demographics, the linear mixed-effect model was reiterated with new fixed factors (other than *musicianship*) to examine the effects of pre- and post-lingual deafness, musical preference (pertaining to genre), and time spent with music listening on SQR.

## Results

### Effect of FRM

**Figure 5.**
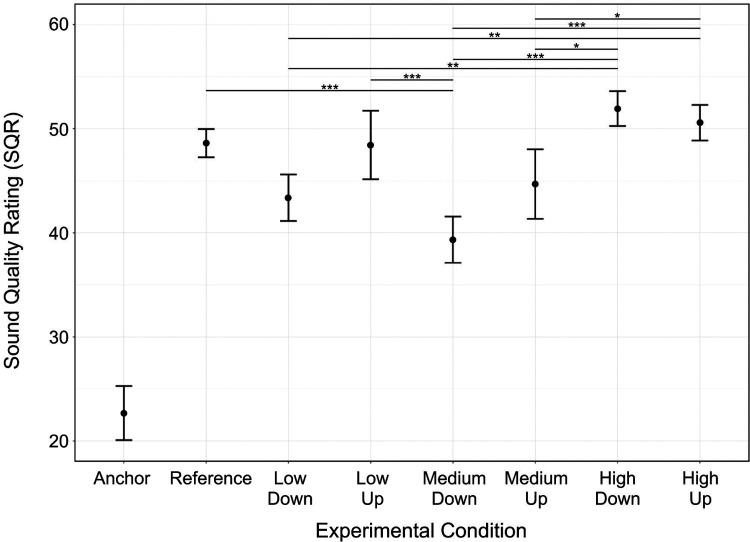
Impact of FRM on MUSHRA ratings. Each dot represents within-participant (*n* *=* *33)* averages across each condition type. Error bars show ±1 standard error from the mean. The Anchor is significantly different from all conditions (*p* *<* *0.001*); these horizontal lines indicating significant differences are omitted for figure clarity. *p *<* 0.05, **p *<* 0.01, ***p *<* *0.001.* Abbreviations: Hz, hertz; MUSHRA, Multiple Stimuli with Hidden Reference and Anchor.

There was a main effect of *condition* [χ^2^(7) = 341.3, *p* < 0.001] (Figure 5). Pairwise comparisons showed that the Anchor led to lower SQR than for any other condition (*p* < 0.001). It was by far the main contributor to this main effect. The Reference led to higher SQR than for MediumDown (*p* < 0.001) but no other comparisons relative to reference were significant (*p* > 0.087). The mean SQRs (±1 standard deviation) for each condition were: Anchor: 23 ± 15; Reference: 49 ± 8; LowDown: 43 ± 13; LowUp: 48 ± 19; MediumDown: 39 ± 13; MediumUp: 45 ± 19; HighDown: 52 ± 10; HighUp: 51 ± 10. Among the six manipulations, the SQR for MediumDown was not different from that for LowDown (*p* = 0.365) or MediumUp *(p* = 0.076) but was lower than for LowUp, HighDown, and HighUp (*p* < 0.001 in all cases). The SQR for HighUp did not differ from that for HighDown (*p* = 0.996), but both SQRs were higher than for MediumUp (*p* < 0.031), MediumDown (*p* < 0.001) and LowDown (*p* < 0.002).

### Effect of Musicianship

**Figure 6.**
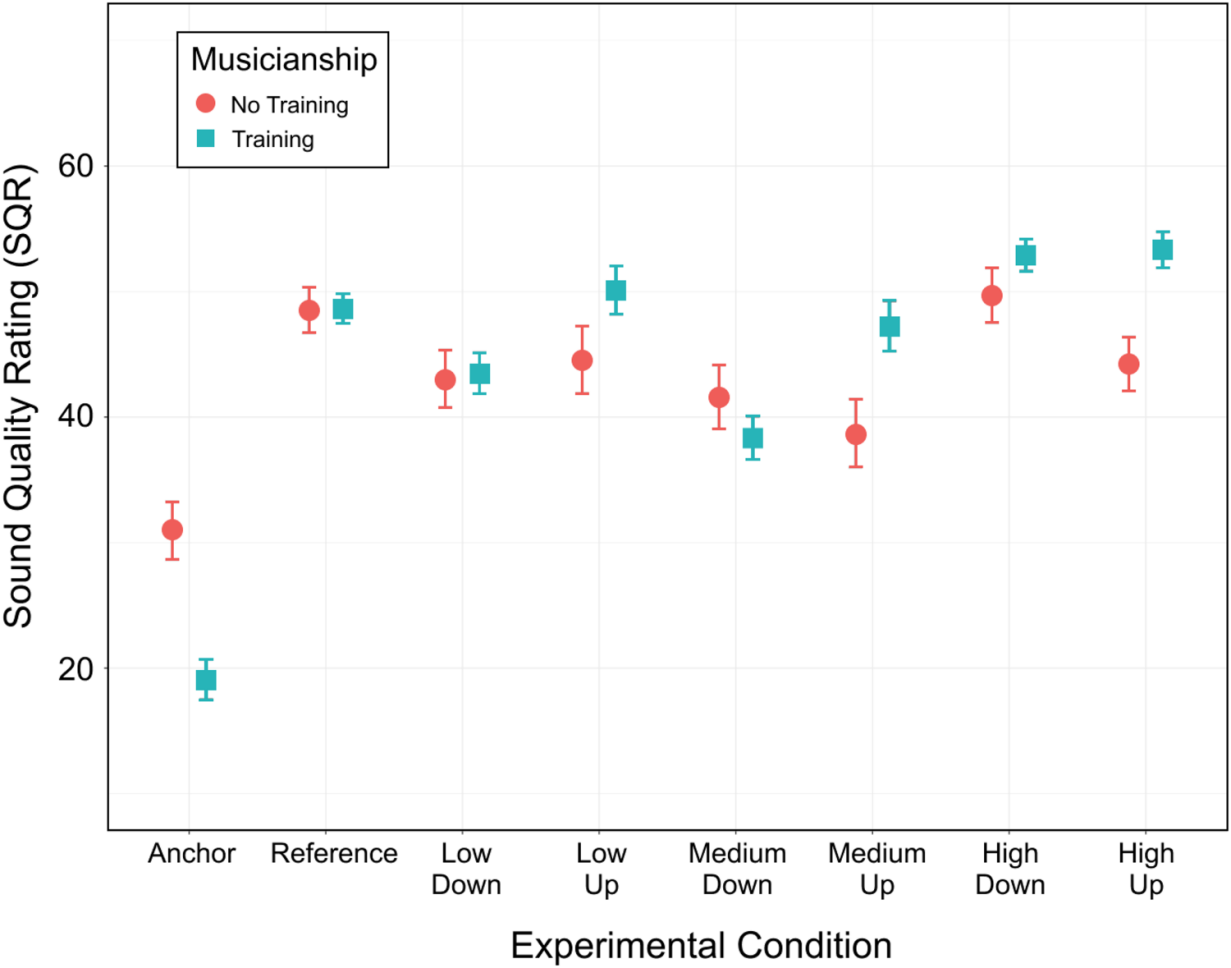
Impact of musical training on SQR for each condition. Participants are split into two groups: those with musical training (*n* *=* *23*) and those without (*n* *=* *10*). Each dot represents within-participant averages for a given condition. Error bars show ±1 standard error from the mean.

In the second approach, *musicianship* did not result in a main effect [χ^2^(1) = 0.4, *p* = 0.526] but it interacted with *condition* [χ^2^(7) = 41.2, *p* < 0.001]. There were simple effects of *musicianship* for Anchor (*p* = 0.001), MediumUp (*p* = 0.018) and HighUp (*p* = 0.012) but not for any other condition (*p* > 0.123). As illustrated in Figure 6, participants without musical training did not rate the Anchor as poorly as musically trained participants, and the former also gave lower ratings for the gain increases of medium and high frequency ranges. Thus, SQR for musically trained participants differed more across conditions, in line with our third hypothesis. The third approach (that ignored Anchor and Reference) revealed a main effect of *frequency range* [χ^2^(2) = 47.6, *p* < 0.001], a main effect of *gain* [χ^2^(1) = 7.7, *p* = 0.006], and an interaction between the two [χ^2^ = 8.1, *p* = 0.018]. The effect of *Musicianship*, once again, was not significant [χ^2^(1) = 1.7, *p* = 0.188], but it interacted with *gain* [χ^2^(1) = 10.3, *p* = 0.001]. No other interaction (2- or 3-way) reached significance (*p* > 0.410). The main effect of *frequency range* and that of *gain* may not be so informative, since the two interacted. This interaction revealed that the effect of *gain* (up > down) occurred for low-frequency and mid-frequency regions (by 5.1 and 5.3 points, respectively, *p* = 0.007 and *p* = 0.005) but not for the high-frequency region (*p* = 0.472), consistent with our first and second hypotheses. Finally, the interaction between *gain* and *musicianship* revealed that musicians preferred up over down stimuli (by 5.3 points, *p* < 0.001) whereas non-musicians did not exhibit this preference (up vs. down, *p* = 0.250).

### Effect of Pre- versus Post-Lingual Deafness

**Figure 7.**
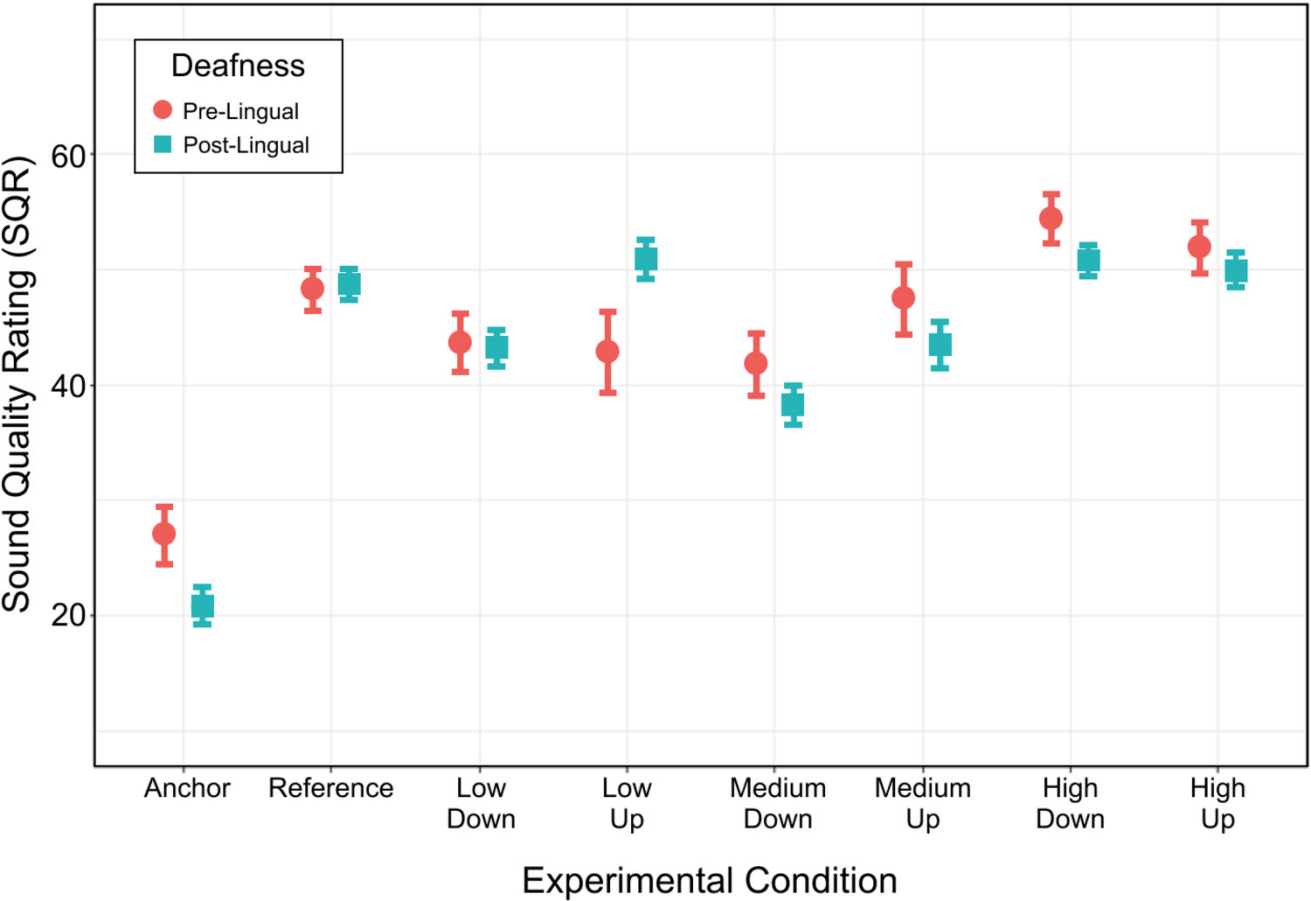
Impact of pre- versus post-lingual deafness on SQR. Participants were split into two groups: those who were pre-lingually deafened (*n* *=* *10)* and those who were post-lingually deafened (*n* *=* *23*). Each dot represents within participant averages for a given condition. Error bars show ±1 standard error from the mean.

In the present study, three out of ten pre-lingual participants did not have musical training. Among the 23 post-lingual participants, there were seven without musical training. A chi-square test revealed that the proportion of musically trained participants was similar for the two groups [χ^2^(1) < 0.1, *p* = 0.980]. A similar analysis was conducted as for *musicianship* but this time with a fixed factor, *lingual*, to determine potential SQR differences between pre- and post-lingually deafened participants (Figure 7). *Lingual* did not result in a main effect [χ^2^(1) = 0.3, *p* = 0.556], but it interacted with condition [χ^2^(7) = 16.4, *p* = 0.022]. To explore this interaction, the Anchor and Reference were discarded to focus on the direction of *gain* and *frequency range*. *Lingual*, again, did not result in a main effect [χ^2^(1) < 0.1, *p* = 0.769], but it interacted with *frequency range* [χ^2^(2) = 8.1, *p* = 0.018] and not with *gain* or in a three-way manner (*p* > 0.172). Post-hoc analyses revealed that pre-lingually deafened participants displayed higher SQR for the manipulations that affected high frequencies than for those at low and medium frequencies (*p* < 0.002), which did not differ from each other (*p* = 0.838). In contrast, post-lingually deafened participants displayed lower SQR for the manipulations that affected the medium range versus those affecting high and low frequencies (*p* < 0.001), which did not differ from each other (*p* = 0.090).

### Effect of Listening Habits

**Figure 8.**
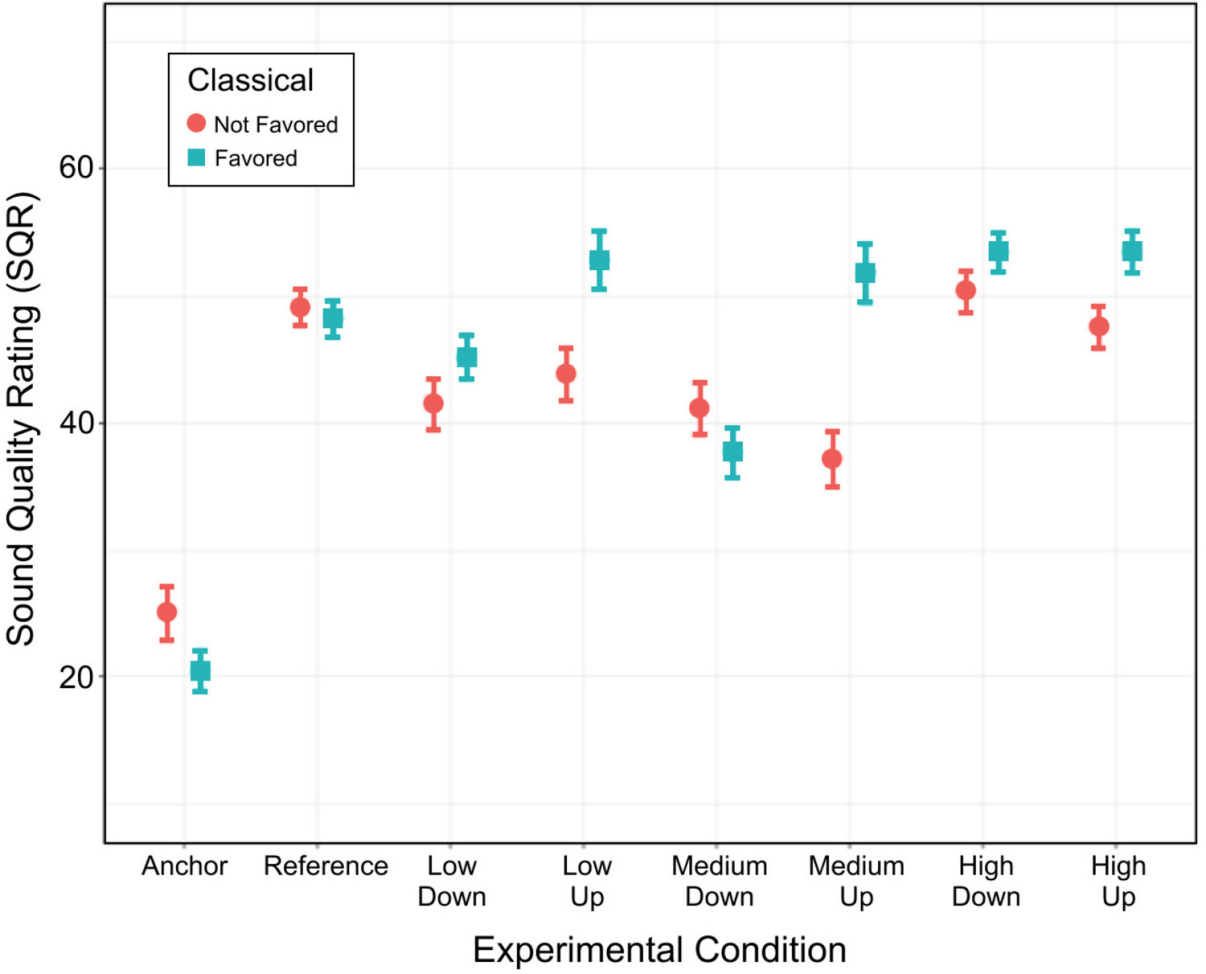
Impact of favoring the classical music genre on SQR. Participants were split into two groups: those who favored classical music (*n* *=* *17)* and those who did not (*n* *=* *16*). Each dot represents within participant averages for a given condition. Error bars show ± 1 standard error from the mean.

The listening habits of our participants demonstrated that the *classical* music genre was their favorite to listen to. To understand whether this was due to the group's relatively high level of musical training, a logistic mixed-effect model (still considering random intercepts by participant) was conducted on the binary listening habit and revealed a main effect of *genre* [χ^2^(10) = 82.3, *p* < 0.001] but no effect of musical training [χ^2^(1) = 0.4, *p* = 0.537] and no interaction [χ^2^(10) = 7.5, *p* = 0.675]. Out of the 33 participants, nine did not listen to *classical* while seven participants selected *classical* along with several other genres. To optimize the sample balance, we normalized participant responses by the total number of genres they listened to. For example, if a participant listened to *classical*, *rock*, and *jazz*, their *classical* “score” was converted to 0.33. An arbitrary cutoff of 0.25 generated a group of 16 “classical non-favorers” versus a group of 17 “classical favorers.” This variable was then entered as a fixed factor, *classical,* similar to *musicianship* or *lingual. Classical* interacted with experimental condition [χ^2^(7) = 43.5, *p* < 0.001]. To further explore this interaction, the analysis was reiterated with the six FRM only and demonstrated a main effect of *classical* [χ^2^(1) = 4.2, *p* = 0.041], which interacted with *gain* [χ^2^(1) = 16.4, *p* < 0.001], and in a 3-way interaction [χ^2^(2) = 9.7, *p* = 0.008] (Figure 8). Participants who did not favor *classical* showed no preference for decreases versus increases of gain for any frequency range (*p* > 0.134), whereas participants who favored *classical* showed a preference for gain increases over decreases at low frequencies (*p* = 0.003, by 7.7 points) and medium frequencies (*p* < 0.001, by 14.2 points) but not at high frequencies (*p* = 0.995). Therefore, these results depicted a similar pattern as for musical training.

One last analysis was conducted on *listening time,* to determine if time spent listening to music affected SQR. A cutoff of six hours per week was used to partition the sample into roughly equal groups. There was no main effect of *listening time* [χ^2^(1) = 0.1, *p* = 0.731] and no interaction with *condition* [χ^2^(7) = 10.5, *p* = 0.164].

## Discussion

FRM affected CI-mediated musical sound quality assessments. Gain increases for low and mid-range frequencies led to higher ratings than reductions of gain by the same amount (9 dB), but not higher ratings than for the reference. These higher ratings may occur because of greater bass and rhythmic sensations, as well as improved vocal comprehension. We caution, however, that the current findings may perhaps not generalize well to all CI users. In this study, many were musically trained, listened to music quite often, and preferred classical music. This profile of CI users is different than that traditionally found in past studies. We found that the results depended on (1) musical training, (2) listening habits, and (3) pre- and post-lingual deafness.

We hypothesized that boosting mid-range frequencies would result in higher CI-mediated sound quality assessments due to the increased salience of vocal information. While a mid-range gain increase did not lead to higher SQR than for the reference, mid-range attenuation reduced sound quality, possibly due to the diminished intelligibility of vocals. Especially for CI users, speech comprehension is an essential component of vocal music listening because it improves song recognition (Gfeller et al., 2008). Lyrics and vocals may act as an auditory “guiderail,” helping CI users to derive more meaning from their listening experience than for music without lyrics (Gfeller, 2009). Beyond the potential for improved song recognition, amplified vocals can help CI users trigger past associations and memories related to a piece of music, which may further improve song comprehension (Gfeller, 2009). In this vein, our finding that increased gain of mid-range frequencies led to higher SQR than for mid-range reductions supports the importance of intelligible vocal cues for CI user musical sound quality. Overall, these results are consistent with past reports that CI users prefer musical stimuli with clear and accentuated vocals (Buyens et al., 2014; Gfeller, 2009; Jiam et al., 2017).

A low frequency gain increase also led to higher SQR than for a gain decrease, but not higher than for the reference. Bass information often provides important rhythmic cues in popular music, contributing substantially to the overall beat of a song. CI users can often perceive simple rhythmic patterns as well as individuals with normal hearing (McDermott, 2004). As a result, CI users often prefer music with prominent and repetitive rhythmic patterns (Jiam et al., 2017). Amplifying bass frequencies may increase the salience of rhythmic and beat information which may lead to higher SQR relative to low-frequency attenuation. Buyens et al. (2014) hypothesized that CI users preferred a louder bass/drum track relative to other background instruments due to a reduction in the perceived subjective complexity of the stimuli. This is relevant because CI user music ratings decrease with increasing perceived complexity (Gfeller et al., 2003). By increasing bass and rhythmic emphasis with low-range gain amplification, CI users may perceive music as more straightforward and simpler to listen to because these aspects of music become more noticeable compared to others. In our study, all musical clips contained higher frequency (>500 Hz) strings, guitar, piano, and/or background vocals, and these often had greater melodic, harmonic, and rhythmic variation than the steadier bass and drum tracks. Low-frequency gain increases may shift the listener's auditory attention to more rhythmic aspects of music, thereby decreasing the overall perceived complexity. Similarly, mid-range gain increases may help reduce music's perceived complexity (for music with vocals) because CI users can more easily attend to speech information, which may be more familiar and intelligible than other musical components.

There were little to no SQR differences due to high-range FRM, suggesting that CI users may not reliably utilize high-frequency information when making sound quality assessments, even though these manipulations may be perceived. In contrast to normal-hearing listeners, CI users gave equal sound quality ratings for a reference clip and the same clip low pass filtered at 4 kHz, suggesting that frequency information above this cutoff contributed little to sound quality (Roy et al., 2012b). Our results are largely in agreement, although our manipulations were less drastic since we only manipulated the gain while Roy et al. effectively removed all audible high-frequency information. It is plausible that our FRM at high frequencies were too subtle to produce sound quality differences, or that these manipulations have no impact on sound quality (i.e., a participant can perceive contrasting timbres but give similar SQR). Greater attenuations of high frequencies might elicit deterioration in musical sound quality but would presumably have to affect the 2–4 kHz range. An alternative explanation as to why significant SQR differences were not observed for high-range FRM is related to our RMS normalization procedure. RMS normalization of the HighUp and HighDown conditions affected the gain at frequencies below 2 kHz. For example, the HighUp condition may in theory have led to improved SQR, but RMS normalization might have offset this benefit due to the corresponding decreased gain of frequencies below 2 kHz, which would worsen sound quality. These cancellation effects may be applicable to other manipulations and represent a limitation of our FRM methodology.

One aim of this study was to determine whether prior musical training alters CI users’ sound quality judgements. As hypothesized, participants with musical training were more sensitive in their SQR than non-trained participants, as evidenced by a significantly lower-rated anchor among this subpopulation. Furthermore, participants with musical training rated increased gain at low and medium frequencies more highly than decreased gain, whereas non-trained participants rated the gain manipulations equally. These results suggest that CI users with a musical background are more attuned to subtle differences in musical stimuli. Multiple studies have shown that post-implantation music training can significantly improve music perception for CI users regardless of prior musical experience (Shukor et al., 2021). While this may be true, we found that pre-implantation musical training was key: 20 of the 23 trained participants completed the vast majority of their musical training prior to cochlear implantation. This suggests that pre-implantation musical training may play a role in helping CI users extract meaningful information from a degraded signal, consistent with a regression analysis by Gfeller et al. (2008). Pre-implantation musical training may additionally yield greater SQR sensitivity because it could prime expectations that small changes can lead to substantial expressive effects. As an example, a more dynamic vocal line (one that has greater gain variations) may be perceived as having more fervent expression and musically trained individuals may make this association more readily than non-trained individuals. Thus, even though a CI user's electric hearing bears little resemblance to their acoustic hearing prior to implantation, musical training may help an individual to perceive auditory subtleties. Overall, this greater sensitivity suggests that CI users with considerable music training could benefit from individualized mapping or from audio applications that allow for listener modifications to optimize musical sound quality.

Notably, the distribution of musical training and preference for our participant sample is atypical when compared with prior studies involving large samples of CI users, including Drennan et al. (2015) and Gfeller et al. (2008). Our sample had unusually high levels of formal music training, above-average time spent with music listening, and genre preferences skewed towards classical music. Thus, the responses of our sample may not be reflective of a more typical population of CI users. Although time spent with music listening had no effect on SQR, there were SQR differences between classical “favorers” and “non-favorers.” Participants who favored classical music showed preferences for gain increases over decreases in the low and medium-range frequencies whereas participants who did not favor classical music demonstrated no preference for each frequency region. Given that listening time did not affect SQR, it may be that participants who are better able to hear subtle sound quality differences are more inclined to listen to classical music.

Pre- and post-lingually deafened participants also differed in how their SQR varied with frequency range. Participants who were post-lingually deafened gave lower SQR for manipulations that affected the medium range as compared with high and low frequency ranges. The lack of a gain interaction makes this effect difficult to interpret. However, following the idea that gain increases in the medium frequency range may be liked for reasons related to speech intelligibility, this result is consistent with the idea that post-lingual users are more prone to judge musical sound quality based on whether lyrics are easier or harder to understand. This itself may be due to differences in speech perception ability between pre- and post-lingually deafened CI users, as post-lingual users usually have better speech intelligibility (Boisvert et al., 2020; Santarelli et al., 2008). Furthermore, it may also be that post-lingually deafened CI users are more sensitive to changes within the speech frequency range, as these users may be more inclined to focus on speech sounds.

There are some limitations to note. Participants were not asked to turn off optional front-end processing features. While we aimed to examine general trends in SQR regardless of participants’ CI manufacturer and features, the use of variable equipment and settings presumably had an impact on SQRs. Future studies should examine how FRM affects musical sound quality with standardized listening modes to better account for variations in listening/processing strategies and dynamic range. Next, all song stimuli used in this study were within the popular music genre. Testing music from other genres that have different structural and acoustical characteristics may yield differing results with the CI-MUSHRA paradigm. Also, it seems reasonable to think that FRM would have more effect for genres that raised more of the participants’ interest to begin with. Perhaps we would have seen larger SQR variations if the original clips were taken from the classical genre. Future studies should explore FRM for a variety of real-world music to determine if the CI-MUSHRA paradigm is sensitive enough to detect potential sound quality differences. Another limitation was that we opted to manipulate gain for wide frequency ranges to examine general trends in SQRs. Our use of broad frequency ranges may have missed potential SQR improvements. Thus, exploring FRM for narrower frequency ranges may demonstrate additional SQR effects.

## Conclusions

The present study explored whether FRM altered musical sound quality for CI users. While no condition led to improved sound quality relative to an unaltered reference clip, CI users’ SQR changed with FRM. Amplification of mid and low-range frequencies resulted in higher SQR than similar reductions of these ranges, at least for a subset of participants who were musically trained and happened to like classical music. Future investigations into more nuanced range and gain manipulations, ideally on an individual and song-level basis, may contribute to improved sound quality and listening experiences for CI users in the future.

## Supplemental Material

sj-png-1-tia-10.1177_23312165221120017 - Supplemental material for Effect of Frequency Response Manipulations on Musical Sound Quality for Cochlear Implant Users Click here for additional data file. Supplemental material, sj-png-1-tia-10.1177_23312165221120017 for Effect of Frequency Response Manipulations on Musical Sound Quality for Cochlear Implant Users by Jonathan Mo, Nicole T. Jiam, Mickael L.D. Deroche, Patpong Jiradejvong and Charles J. Limb in Trends in Hearing

sj-docx-2-tia-10.1177_23312165221120017 - Supplemental material for Effect of Frequency Response Manipulations on Musical Sound Quality for Cochlear Implant Users Click here for additional data file. Supplemental material, sj-docx-2-tia-10.1177_23312165221120017 for Effect of Frequency Response Manipulations on Musical Sound Quality for Cochlear Implant Users by Jonathan Mo, Nicole T. Jiam, Mickael L.D. Deroche, Patpong Jiradejvong and Charles J. Limb in Trends in Hearing
