# Supplementary figures and images for: Effect of Frequency Response Manipulations on Musical Sound Quality for Cochlear Implant Users
Source: Trends Hear. 2022 Aug 19;26:23312165221120017. doi: 10.1177/23312165221120017 (PMC9393940; doi:10.1177/23312165221120017)

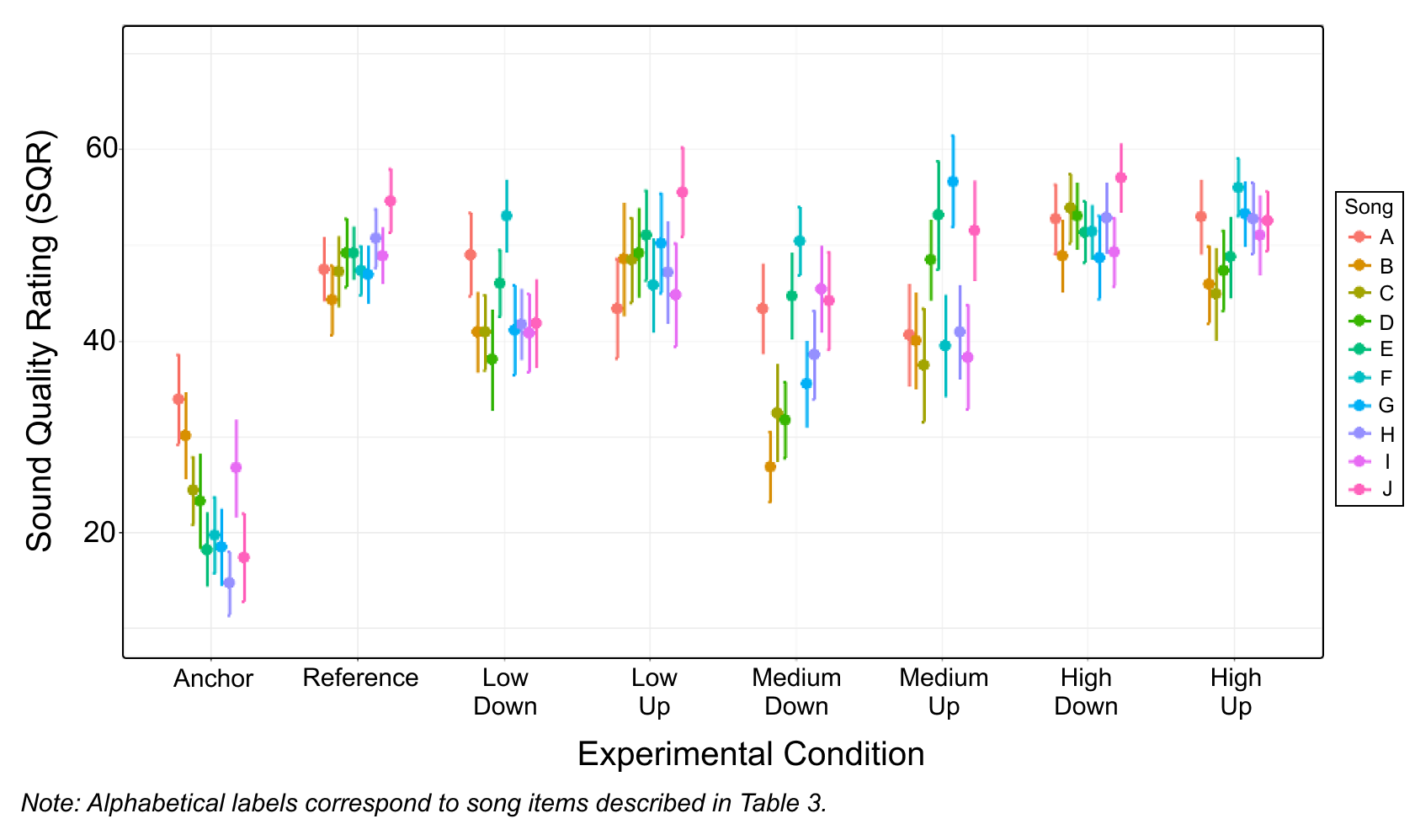

Supplement: sj-png-1-tia-10.1177_23312165221120017 - Supplemental material for Effect of Frequency Response Manipulations on Musical Sound Quality for Cochlear Implant Users [file sj-png-1-tia-10.1177_23312165221120017.png]
