# Supplementary material for: Effect of Frequency Response Manipulations on Musical Sound Quality for Cochlear Implant Users
Source: Trends Hear. 2022 Aug 19;26:23312165221120017. doi: 10.1177/23312165221120017 (PMC9393940; doi:10.1177/23312165221120017)
Supplement: sj-docx-2-tia-10.1177_23312165221120017 - Supplemental material for Effect of Frequency Response Manipulations on Musical Sound Quality for Cochlear Implant Users [file sj-docx-2-tia-10.1177_23312165221120017.docx]

UCSF Frequency Equalization Study

Start of Block: Welcome/Pre-screening Block

Q0


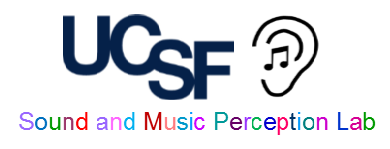


Q1
**Sound and Music Perception Lab at UCSF**

We are conducting research study regarding music perception in cochlear implant users. We invite all cochlear implant users to participate. Your responses will be kept confidential and aggregated to ensure anonymity. **This survey should take you 30 to 60 minutes.**

If you have any questions about the study, please direct them to the Principal Investigators: Charles Limb, MD (Charles.Limb@ucsf.edu) or Nicole Jiam, MD (Nicole.Jiam@ucsf.edu). If you wish to ask questions about the study or your rights as a research participant to someone other than the researchers or if you wish to voice any problems or concerns you may have about the study, please call the UCSF Institutional Review Board at 415-476-1814.   

Q2 Please select one of the choices below:

- Younger than 18 years old (1)
- 18+ years of age (2)

Q3 Do you currently use a cochlear implant?

- Yes (1)
- No (2)

Display This Question:

If Please select one of the choices below: = Younger than 18 years old

Or Do you currently use a cochlear implant? = No

Q4 Thank you for your interest and time! Unfortunately, we require that all participants in this study are 18+ years of age and currently use a cochlear implant.

Skip To: End of Survey If Thank you for your interest and time! Unfortunately, we require that all participants in this stu... Is Displayed

End of Block: Welcome/Pre-screening Block

Start of Block: Direct Connect Questionnaire

Q5 **Direct Connect Questionnaire**   In order to determine your eligibility of the study, we need to make sure you have the capability to use direct connect with your cochlear implant while participating in the study tasks. If you have any questions regarding your direct connect system, please email Nicole.Jiam@ucsf.edu.

Q6 What kind of cochlear implant do you have?

- Advanced Bionics (1)
- Cochlear Americas (2)
- MED-EL (3)

Display This Question:

If What kind of cochlear implant do you have? = Advanced Bionics

Q7 Do you have one of the following direct connection options?

- CI Connect (attaches to the standard PowerCel 170) (3)
- Roger Select Transmitter (4)
- Roger Pen Transmitter (5)
- ComPilot I (6)
- None of the Above (7)

Display This Question:

If Do you have one of the following direct connection options? = Roger Select Transmitter

Or Do you have one of the following direct connection options? = Roger Pen Transmitter

Q8 Do you also have one of the following additional accessories?

- Roger 17 receiver (that attaches to the standard PowerCel 170) (1)
- Roger X receiver (plugs into ComPilot) (2)
- Roger MyLink (induction to Telecoil program on processor) (3)
- Audio Cable (4)
- None of the Above (5)

Display This Question:

If Do you have one of the following direct connection options? = None of the Above

Q9 Unfortunately you do not have the necessary equipment to take part in this study.
You may now exit this survey. We sincerely thank you for your time!

Skip To: End of Survey If Unfortunately you do not have the necessary equipment to take part in this study. You may now exi... Is Displayed

Display This Question:

If Do you also have one of the following additional accessories? = None of the Above

Q10 Unfortunately you do not have the necessary equipment to take part in this study.
You may now exit this survey. We sincerely thank you for your time!

Skip To: End of Survey If Unfortunately you do not have the necessary equipment to take part in this study. You may now exi... Is Displayed

Display This Question:

If Do you have one of the following direct connection options? = CI Connect (attaches to the standard PowerCel 170)

Or Do you have one of the following direct connection options? = Roger Select Transmitter

And Do you also have one of the following additional accessories? = Roger 17 receiver (that attaches to the standard PowerCel 170)

Or Do you have one of the following direct connection options? = Roger Select Transmitter

And Do you also have one of the following additional accessories? = Roger X receiver (plugs into ComPilot)

Or Do you have one of the following direct connection options? = Roger Select Transmitter

And Do you also have one of the following additional accessories? = Roger MyLink (induction to Telecoil program on processor)

Or Do you have one of the following direct connection options? = Roger Select Transmitter

And Do you also have one of the following additional accessories? = Audio Cable

Or Do you have one of the following direct connection options? = Roger Pen Transmitter

And Do you also have one of the following additional accessories? = Roger 17 receiver (that attaches to the standard PowerCel 170)

Or Do you have one of the following direct connection options? = Roger Pen Transmitter

And Do you also have one of the following additional accessories? = Roger X receiver (plugs into ComPilot)

Or Do you have one of the following direct connection options? = Roger Pen Transmitter

And Do you also have one of the following additional accessories? = Roger MyLink (induction to Telecoil program on processor)

Or Do you have one of the following direct connection options? = Roger Pen Transmitter

And Do you also have one of the following additional accessories? = Audio Cable

Or Do you have one of the following direct connection options? = ComPilot I

Q11 FOR ADVANCED BIONICS COCHLEAR IMPLANT USERS:

Based on your answers, we believe you have the necessary equipment to participate in this study. Please use your direct connect equipment and connect it now to the device you are using to take this survey (e.g. computer, tablet, phone). We want to make sure that any of the sound that would typically be playing from the device is directly transmitted to your cochlear implant. Please feel free to use the quick reference sheet below or to email nicole.jiam@ucsf.edu with any questions:

*Option 1:* You may use your CI Connect (attaches to standard PowerCel 170) and pair it to any bluetooth-equipped device. This option is compatible with Naida Q90.

*Option 2:* You may use your Roger Select or Pen transmitter (pairs to bluetooth-equipped device) and one of the following: Roger 17 receiver (attaches to standard PowerCel 170), Roger X receiver (plugs into ComPilot), Roger MyLink (induction to Telecoil program on processor). Transmitters may also be plugged into standard 3.5mm headphone jack with an audio cable if the device is not bluetooth equipped. This option is compatible with Naida Q90 and Q70.

*Option 3:* You may use your ComPilot I to pair to bluetooth-equipped devices or plug into the headphone jack of audio source (with an audio cable). This device is worn around patient’s neck and compatible with Naida Q90 and Q70.

Note: The program that is being be used must have the Roger and/or ComPilot feature turned on. Please make sure that the mixing ratio is using entirely streamed and not using any input from the  environmental microphone.

Display This Question:

If What kind of cochlear implant do you have? = Cochlear Americas

Q12 Do you have one of the following direct connection options?

- Phone Clip (1)
- MiniMicrophone 2+ (2)
- iPad or iPhone (3)
- None of the Above (4)

Display This Question:

If Do you have one of the following direct connection options? = None of the Above

Q13 Unfortunately you do not have the necessary equipment to take part in this study.
You may now exit this survey. We sincerely thank you for your time!

Skip To: End of Survey If Unfortunately you do not have the necessary equipment to take part in this study. You may now exi... Is Displayed

Display This Question:

If Do you have one of the following direct connection options? = Phone Clip

Or Do you have one of the following direct connection options? = MiniMicrophone 2+

Or Do you have one of the following direct connection options? = iPad or iPhone

Q14 FOR COCHLEAR AMERICAS COCHLEAR IMPLANT USERS:

Based on your answers, we believe you have the necessary equipment to participate in this study. Please use your direct connect equipment and connect it now to the device you are using to take this survey (e.g. computer, tablet, phone). We want to make sure that any of the sound that would typically be playing from the device is directly transmitted to your cochlear implant. Please feel free to use the quick reference sheet below or to email nicole.jiam@ucsf.edu with any questions:

*Option 1:* You may use your Phone Clip to pair with a bluetooth-equipped device to directly stream to your cochlear implant processor. This option is compatible with Kanso 2, N7, Kanso, and N6.

*Option 2:* You may use your MiniMicrophone 2+ and plug it into a 3.5mm headphone jack with an audio cable. This option is compatible with Kanso 2, N7, Kanso, and N6. *Option 3:* If you are using an iPad and iPhone, you can stream directly to your Kanso 2 or N7.

Display This Question:

If What kind of cochlear implant do you have? = MED-EL

Q15 Do you have one of the following direct connection options?

- AudioLink (1)
- Roger Select Transmitter (2)
- Roger Pen Transmitter (3)
- None of the Above (4)

Display This Question:

If Do you have one of the following direct connection options? = Roger Select Transmitter

Or Do you have one of the following direct connection options? = Roger Pen Transmitter

Q16 Do you also have one of the following additional accessories?

- Roger 21 receiver (that is built-into Sonnet/Sonnet 2 battery cover) (1)
- Roger X receiver (plugs into Opus 2 FM battery cover) (2)
- Roger MyLink (induction to Telecoil program on processor) (3)
- Audio Cable (4)
- None of the Above (6)

Display This Question:

If Do you have one of the following direct connection options? = None of the Above

Q17 Unfortunately you do not have the necessary equipment to take part in this study.
You may now exit this survey. We sincerely thank you for your time!

Skip To: End of Survey If Unfortunately you do not have the necessary equipment to take part in this study. You may now exi... Is Displayed

Display This Question:

If Do you also have one of the following additional accessories? = None of the Above

Q18 Unfortunately you do not have the necessary equipment to take part in this study.
You may now exit this survey. We sincerely thank you for your time!

Skip To: End of Survey If Unfortunately you do not have the necessary equipment to take part in this study. You may now exi... Is Displayed

Display This Question:

If Do you have one of the following direct connection options? = AudioLink

Or Do you have one of the following direct connection options? = Roger Select Transmitter

And Do you also have one of the following additional accessories? = Roger 21 receiver (that is built-into Sonnet/Sonnet 2 battery cover)

Or Do you have one of the following direct connection options? = Roger Select Transmitter

And Do you also have one of the following additional accessories? = Roger X receiver (plugs into Opus 2 FM battery cover)

Or Do you have one of the following direct connection options? = Roger Select Transmitter

And Do you also have one of the following additional accessories? = Roger MyLink (induction to Telecoil program on processor)

Or Do you have one of the following direct connection options? = Roger Select Transmitter

And Do you also have one of the following additional accessories? = Audio Cable

Or Do you have one of the following direct connection options? = Roger Pen Transmitter

And Do you also have one of the following additional accessories? = Roger 21 receiver (that is built-into Sonnet/Sonnet 2 battery cover)

Or Do you have one of the following direct connection options? = Roger Pen Transmitter

And Do you also have one of the following additional accessories? = Roger X receiver (plugs into Opus 2 FM battery cover)

Or Do you have one of the following direct connection options? = Roger Pen Transmitter

And Do you also have one of the following additional accessories? = Roger MyLink (induction to Telecoil program on processor)

Or Do you have one of the following direct connection options? = Roger Pen Transmitter

And Do you also have one of the following additional accessories? = Audio Cable

Q19 FOR MED-EL COCHLEAR IMPLANT USERS:

Based on your answers, we believe you have the necessary equipment to participate in this study. Please use your direct connect equipment and connect it now to the device you are using to take this survey (e.g. computer, tablet, phone). We want to make sure that any of the sound that would typically be playing from the device is directly transmitted to your cochlear implant. Please feel free to use the quick reference sheet below or to email nicole.jiam@ucsf.edu with any questions:

*Option 1:* You may use your AudioLink to pair with your bluetooth-equipped devices. Alternatively, your AudioLink could also be directly plugged to the device using an 3.5mm audio cable. This option is compatible with Sonnet 2 and will work with Sonnet as long as the you also have the AudioKey app.

*Option 2:* You may use your Roger Select or Pen transmitter (pairs to BT-equipped device) and one of the following: Roger 21 receiver that is built-into Sonnet/Sonnet 2 battery cover, Roger X receiver plugged into Opus 2 FM battery cover, or Roger MyLink (induction to Telecoil program on processor). Transmitters may alternatively be plugged into the standard 3.5mm headphone jack with an audio cable if the device is not bluetooth equipped. This option is compatible with Sonnet 2, Sonnet, and Rondo.

End of Block: Direct Connect Questionnaire

Start of Block: Consent Block (1/2)

Q20
Before continuing with this study, it is important that you **review the following consent form**that will inform you about several tasks that you will be participating in.
 
Please read and sign the **Consent Form** found on the next page.

End of Block: Consent Block (1/2)

Start of Block: Consent Block (2/2)

Q21 UNIVERSITY OF CALIFORNIA, SAN FRANCISCO CONSENT TO PARTICIPATE IN A RESEARCH STUDY
  
**Study Title:** Cochlear implant-mediated perception of music

This is a research study investigating music and complex sound perception in cochlear implant users. The study principal investigator, Charles Limb, MD, and/or study coordinator from the UCSF Department of Otolaryngology will explain this study to you.

Research studies include only people who choose to take part. Please take your time to make your decision about participating, and discuss your decision with your family or friends if you wish. If you have any questions, you may ask the researchers.

You are being asked to take part in this study either as a cochlear implant patient or as a healthy volunteer.

**Why is this study being done?**

Because of music’s complexity, music perception in people with cochlear implants (surgically- implanted hearing aids) can be difficult. The purpose of this study is to learn about the way that individuals with cochlear implants hear music differently from people without hearing aids. This study is funded by the UCSF Department of Otolaryngology and the Boys Town National Research Hospital. One arm of this study is funded by Advanced Bionics, LLC.

**How many people will take part in this study?**

Approximately 400 people will take part in this study.

**What will happen if I take part in this research study?**

- You will be asked to complete a few short questionnaires. For example, these might gather information about your cochlear implant (if you are a cochlear implant recipient) and your level of musical training. You might also be asked to participate in a few cognitive tests.
- You will be asked to complete a short hearing test in which you will listen to a series of musical excerpts and answer a few response questions about each. You may also be asked to verbally reproduce sounds, sing simple songs, or read some words. In some cases, you’ll be asked to participate in these hearing and sound tests using modified processing strategies.
- Additionally, an electroencephalogram (EEG) may be used to non-invasively record your electrical brain activity while you hear or produce music or sound. During an EEG exam, small sensors are attached to the surface of the scalp using special paste. These sensors on the cortical cap pick up electrical signals produced by brain activity. EEG recordings in this study will take about 20-60 minutes.

**Study location:** These procedures will be done remotely or at either the UCSF Parnassus, Mission Bay, or Mount Zion campus.

**How long will I be in the study?**

Participation in the study will take approximately 1-4 hours.

**Can I stop being in the study?**

Yes. You can decide to stop at any time. Just tell the study researcher or staff person right away if you wish to stop being in the study. Also, the study researcher may stop you from taking part in this study at any time if he or she believes it is in your best interest, if you do not follow the study rules, or if the study is stopped.

**What side effects or risks can I expect from being in the study**?

This is a minimum-risk study. There is a small risk of loss of confidentiality of your private information; however, we will do everything possible to ensure that this does not happen. All of your data and information will be kept in a locked file cabinet in a locked office or in a password-protected electronic database and accessible only by the study team. If you are asked to perform hearing or sound tests using modified processing strategies, the new strategy may produce uncomfortable sounds. However, we will work with you carefully to ensure this does not happen.

**Are there benefits to taking part in the study?**

There will be no direct benefit to you from participating in this study. However, the information that you provide may help health professionals better understand/learn more about music perception in cochlear implant users. The results from this study may help inform cochlear implant design in the future.

**What other choices do I have if I do not take part in this study?**

You are free to choose not to participate in the study. If you decide not to take part in this study, there will be no penalty to you. You will not lose any of your regular benefits, and you can still get your care from our institution the way you usually do.

**Will information about me be kept private?**
 We will do our best to make sure that the personal information gathered for this study is kept private. However, we cannot guarantee total privacy. Your personal information may be given out if required by law. If information from this study is published or presented at scientific meetings, your name and other personal information will not be used.

Organizations that may look at and/or copy your research records for research, quality assurance, and data analysis include:

- The University of California
- Boys Town National Research Hospital

Your information and/or data from this study may be shared with your audiologist or normal care provider if medically relevant. For the arm of the study funded by Advanced Bionics, de- identified data may be shared.

Participation in research may involve a loss of privacy, but information about you will be handled as confidentially as possible. A medical record will be created because of your participation in this study. Your consent form and some of your research test results will be included in this record. Therefore, your other health care providers may see your test results and become aware of your participation. Hospital regulations require that all health care providers treat information in medical records confidentially.

**What are the costs of taking part in this study?**

You will not be charged for any of the study treatments or procedures.

**Will I be paid for taking part in this study?**
There is no payment for **online/remote** studies.

For those completing **in-person** studies, you will be paid $15-75 for taking part in this study, depending on the length of time it takes to complete all study activities. Specifically:

- Less than 1 hour = $15
- 1-2 hours = $30
- 2-3 hours = $45
- 3-4 hours = $60
- More than 4 hours = $75

If applicable, your parking and other travel expenses will also be reimbursed.

**What are my rights if I take part in this study?**

Taking part in this study is your choice. You may choose either to take part or not to take part in the study. If you decide to take part in this study, you may leave the study at any time. No matter what decision you make, there will be no penalty to you in any way. You will not lose any of your regular benefits, and you can still get your care from our institution the way you usually do.

**Who can answer my questions about the study?**

You can talk to the researcher about any questions, concerns, or complaints you have about this study. Contact the researcher Charles Limb, MD at (415) 353-2870.

If you wish to ask questions about the study or your rights as a research participant to someone other than the researchers or if you wish to voice any problems or concerns you may have about the study, please call the Office of the Committee on Human Research at 415-476-1814.

**CONSENT**

You have been given a copy of this consent form to keep.

PARTICIPATION IN RESEARCH IS VOLUNTARY. You have the right to decline to be in this study, or to withdraw from it at any point without penalty or loss of benefits to which you are otherwise entitled. If you wish to participate in this study, you should sign below.

Q22 Select Today's Date:

________________________________________________________________

Q23 Participant's Signature for Consent:
(Click-Hold & Sign)

Q24 **Person Obtaining Consent:** Nicole Jiam, MD

End of Block: Consent Block (2/2)

Start of Block: CI Questionnaire Prompt

Q25 The following set of questions will ask about your **cochlear implant(s) and related hearing history**.

End of Block: CI Questionnaire Prompt

Start of Block: Cochlear Implant Questionnaire

Q26 What is your gender?

- Male (1)
- Female (2)

Q27 How old are you?

________________________________________________________________

Q28 What is the cause of your hearing loss? Select all that apply.

- Born deaf (Congenital) (1)
- Aging (2)
- Noise Exposure (3)
- Meniere’s Disease (4)
- Autoimmune Condition (5)
- Otosclerosis (6)
- Infection (7)
- Antibiotics (8)
- Unknown (9)
- Other (10) ________________________________________________

Q29 How old were you when your hearing loss occurred?

________________________________________________________________

Q30 Have you been diagnosed with **profound** hearing loss?

- Yes (1)
- No (2)

Display This Question:

If Have you been diagnosed with profound hearing loss? = Yes

Q31 How old were you when you developed **profound** hearing loss?

________________________________________________________________

Q32 Did you hearing loss occur before or after you learned to speak?

- Before (1)
- After (2)

Q33 Which side were you implanted on?

- Left ear (1)
- Right ear (2)
- Both ears (3)

Display This Question:

If Which side were you implanted on? = Left ear

Or Which side were you implanted on? = Both ears

Q34 What was the date of **left** cochlear implantation surgery (MM/DD/YYYY)?

________________________________________________________________

Display This Question:

If Which side were you implanted on? = Left ear

Or Which side were you implanted on? = Both ears

Q35 Who is the manufacturer for your **left** cochlear implant?

- Cochlear Americas (1)
- MED-EL (2)
- Advanced Bionics (3)

Display This Question:

If Who is the manufacturer for your left cochlear implant? = Cochlear Americas

Q36 What type of implant do you have for your left cochlear implant?

- Nucleus Profile Plus with Slim Modiolar Electrode (CI632) (5)
- Nucleus Profile Plus with Contour Advance (CI612) (6)
- Nucleus Profile Plus with Slim Straight Electrode (CI622) (7)
- Nucleus Profile with Slim Modiolar Electrode (CI532) (8)
- Nucleus Profile with Contour Advance (CI512) (9)
- Nucleus Profile with Slim Straight Electrode (CI522) (10)
- Nucleus CI422 with Slim Straight Electrode (11)
- Nucleus CI24RE(CA) Contour Advance (12)
- Nucleus CI24RE(ST) Straight (13)
- Nucleus Hybrid L24 Implant (14)
- Other (15) ________________________________________________
- I don’t know (16)

Display This Question:

If Who is the manufacturer for your left cochlear implant? = Cochlear Americas

Q37 What type of sound processor do you have for your left cochlear implant?

- Nucleus 6 (4)
- Nucleus 7 (5)
- Kanso (6)
- Kanso 2 (7)
- Other (8) ________________________________________________
- I don’t know (9)

Display This Question:

If Who is the manufacturer for your left cochlear implant? = MED-EL

Q38 What type of implant do you have for your left cochlear implant?

- Synchrony (4)
- Synchrony 2 (5)
- Other (6) ________________________________________________
- I don’t know (7)

Display This Question:

If Who is the manufacturer for your left cochlear implant? = MED-EL

Q39 What type of electrode array do you have for your left cochlear implant?

- FLEX SOFT (4)
- FLEX 28 (5)
- FLEX 24 (6)
- FLEX 20 (7)
- FORM 24 (8)
- FORM 19 (9)
- Standard (10)
- Medium (11)
- Compressed (12)
- Other (13) ________________________________________________
- I don’t know (14)

Display This Question:

If Who is the manufacturer for your left cochlear implant? = MED-EL

Q40 What type of sound processor do you have for your left cochlear implant?

- SONNET (4)
- SONNET 2 (5)
- RONDO (6)
- RONDO 2 (7)
- OPUS 2 (8)
- Other (9) ________________________________________________
- I don’t know (10)

Display This Question:

If Who is the manufacturer for your left cochlear implant? = Advanced Bionics

Q41 What type of implant do you have for your left cochlear implant?

- HiRes Ultra 3D Implant (4)
- HiRes Ultra Implant (5)
- HiRes 90K (6)
- Other (7) ________________________________________________
- I don’t know (8)

Display This Question:

If Who is the manufacturer for your left cochlear implant? = Advanced Bionics

Q42 What type of electrode array do you have for your left cochlear implant?

- HiFocus SlimJ Electrode (4)
- HiFocus Mid-Scala Electrode (5)
- HiFocus 1J (6)
- HiFocus Helix (7)
- Other (8) ________________________________________________
- I don’t know (9)

Display This Question:

If Who is the manufacturer for your left cochlear implant? = Advanced Bionics

Q43 What type of sound processor do you have for your left cochlear implant?

- Naida CI (4)
- Neptune (5)
- Harmony (6)
- Other (7) ________________________________________________
- I don’t know (8)

Display This Question:

If Which side were you implanted on? = Right ear

Or Which side were you implanted on? = Both ears

Q44 What was the date of right cochlear implantation surgery (MM/DD/YYYY)?

________________________________________________________________

Display This Question:

If Which side were you implanted on? = Right ear

Or Which side were you implanted on? = Both ears

Q45 Who is the manufacturer for your **r**ight cochlear implant?

- Cochlear Americas (1)
- MED-EL (2)
- Advanced Bionics (3)

Display This Question:

If Who is the manufacturer for your right cochlear implant? = Cochlear Americas

Q46 What type of implant do you have for your right cochlear implant?

- Nucleus Profile Plus with Slim Modiolar Electrode (CI632) (4)
- Nucleus Profile Plus with Contour Advance (CI612) (5)
- Nucleus Profile Plus with Slim Straight Electrode (CI622) (6)
- Nucleus Profile with Slim Modiolar Electrode (CI532) (7)
- Nucleus Profile with Contour Advance (CI512) (8)
- Nucleus Profile with Slim Straight Electrode (CI522) (9)
- Nucleus CI422 with Slim Straight Electrode (10)
- Nucleus CI24RE(CA) Contour Advance (11)
- Nucleus CI24RE(ST) Straight (12)
- Nucleus Hybrid L24 Implant (13)
- Other (14) ________________________________________________
- I don’t know (15)

Display This Question:

If Who is the manufacturer for your right cochlear implant? = Cochlear Americas

Q47 What type of sound processor do you have for your **right** cochlear implant?

- Nucleus 6 (4)
- Nucleus 7 (5)
- Kanso (6)
- Kanso 2 (7)
- Other (8) ________________________________________________
- I don’t know (9)

Display This Question:

If Who is the manufacturer for your right cochlear implant? = MED-EL

Q48 What type of implant do you have for your right cochlear implant?

- Synchrony (4)
- Synchrony 2 (5)
- Other (6) ________________________________________________
- I don’t know (7)

Display This Question:

If Who is the manufacturer for your right cochlear implant? = MED-EL

Q49 What type of electrode array do you have for your right cochlear implant?

- FLEX SOFT (4)
- FLEX 28 (5)
- FLEX 24 (6)
- FLEX 20 (7)
- FORM 24 (8)
- FORM 19 (9)
- Standard (10)
- Medium (11)
- Compressed (12)
- Other (13) ________________________________________________
- I don’t know (14)

Display This Question:

If Who is the manufacturer for your right cochlear implant? = MED-EL

Q50 What type of sound processor do you have for your right cochlear implant?

- SONNET (4)
- SONNET 2 (5)
- RONDO (6)
- RONDO 2 (7)
- OPUS 2 (8)
- Other (9) ________________________________________________
- I don’t know (10)

Display This Question:

If Who is the manufacturer for your right cochlear implant? = Advanced Bionics

Q51 What type of implant do you have for your right cochlear implant?

- HiRes Ultra 3D Implant (4)
- HiRes Ultra Implant (5)
- HiRes 90K (6)
- Other (7) ________________________________________________
- I don’t know (8)

Display This Question:

If Who is the manufacturer for your right cochlear implant? = Advanced Bionics

Q52 What type of electrode array do you have for your right cochlear implant?

- HiFocus SlimJ Electrode (4)
- HiFocus Mid-Scala Electrode (5)
- HiFocus 1J (6)
- HiFocus Helix (7)
- Other (8) ________________________________________________
- I don’t know (9)

Display This Question:

If Who is the manufacturer for your right cochlear implant? = Advanced Bionics

Q53 What type of sound processor do you have for your right cochlear implant?

- Naida CI (4)
- Neptune (5)
- Harmony (6)
- Other (7) ________________________________________________
- I don’t know (8)

Q54 Is there anything else you'd like us to know about your cochlear implant(s)? If you are a bilateral cochlear implant user, please indicate which ear is your better hearing ear.

________________________________________________________________

End of Block: Cochlear Implant Questionnaire

Start of Block: Musical Experience Prompt

Q55 The second set of questions will ask you about your **musical experience.**

End of Block: Musical Experience Prompt

Start of Block: Musical Experience Questionnaire

Q56 Which of the following best describes your musical training? Choose the most applicable answer.

- No musical training (either formal or self-taught) (1)
- Self-taught musician (2)
- Formal musical training (3)

Display This Question:

If Which of the following best describes your musical training? Choose the most applicable answer. = Self-taught musician

Or Which of the following best describes your musical training? Choose the most applicable answer. = Formal musical training

Q57 What age did your training start (either self-taught or formal training)?

________________________________________________________________

Display This Question:

If Which of the following best describes your musical training? Choose the most applicable answer. = Self-taught musician

Or Which of the following best describes your musical training? Choose the most applicable answer. = Formal musical training

Q58 How long have you been musically trained (in years)?

________________________________________________________________

Display This Question:

If Which of the following best describes your musical training? Choose the most applicable answer. = Self-taught musician

Or Which of the following best describes your musical training? Choose the most applicable answer. = Formal musical training

Q59 What type of learning environment did you use? Check all that apply.

- Self-taught (by ear) (1)
- Self-taught (by books) (2)
- Online course or videos (3)
- Formal lessons (4)
- Group classes (5)
- Music degree (6)
- Other (7) ________________________________________________

Display This Question:

If Which of the following best describes your musical training? Choose the most applicable answer. = Self-taught musician

Or Which of the following best describes your musical training? Choose the most applicable answer. = Formal musical training

Q60 Are you currently practicing?

- Yes (1)
- No (2)

Display This Question:

If Are you currently practicing? = No

Q61 At what age did you stop your music training/practicing?

________________________________________________________________

Display This Question:

If Which of the following best describes your musical training? Choose the most applicable answer. = Self-taught musician

Or Which of the following best describes your musical training? Choose the most applicable answer. = Formal musical training

Q62 What is the average number of hours per week spent practicing?

________________________________________________________________

Display This Question:

If Which of the following best describes your musical training? Choose the most applicable answer. = Self-taught musician

Or Which of the following best describes your musical training? Choose the most applicable answer. = Formal musical training

Q63 What was your primary instrument or related skill?

________________________________________________________________

Display This Question:

If Which of the following best describes your musical training? Choose the most applicable answer. = Self-taught musician

Or Which of the following best describes your musical training? Choose the most applicable answer. = Formal musical training

Q64 What was your minor instrument or related skill? *If not applicable, please leave this text box blank.*

________________________________________________________________

Q65 What is the average number of hours per week spent listening to music?

________________________________________________________________

Q66 What are your favorite genres of music to listen to? Check all that apply.

- Classical (1)
- Country (2)
- Electronic (3)
- Folk (4)
- Hip-Hop (5)
- Jazz (6)
- Pop (7)
- Rap (8)
- Rhythm and Blues (9)
- Rock (10)
- Metal (11)
- Other (12) ________________________________________________

End of Block: Musical Experience Questionnaire

Start of Block: MUSHRA Tests

| 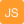 |
| --- |

Q67 **Testing Instructions:**
 
If you have not already, please set up your direct connect equipment so that any sounds from this questionnaire will be directly transmitted to your cochlear implant rather than through the speakers of your device.

If you are a bilateral cochlear implant user, please take the test using your better hearing ear.
 
In the next several questions, we will present you with 20 sets of modified audio clips of a particular song and ask you to compare it to the "reference" song. **Please rank the sound quality of each audio clip from a scale of 0 to 100 in comparison to the reference song. ("0" being a significantly worse sounding than the reference and "100" being a significantly better sounding the reference).** You must rank at least one audio clip at "0" to proceed forward. Please make your judgements based on your initial reaction ("gut feeling"). We do not encourage you to repeat playing the stimuli multiple times.

Before proceeding, please watch this [walkthrough video](https://youtu.be/TI7lr5QdQCo) on how to navigate through the testing interface. In case auto-captioning does not turn on automatically, please click the "CC" button on the bottom right of the video.


(Alternatively, copy and paste this link in a new tab: https://youtu.be/TI7lr5QdQCo)

End of Block: MUSHRA Tests

Start of Block: A

| 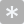 | 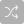 | 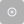 |
| --- | --- | --- |

Q68 Play Reference
A-VB

|  | Significantly worse than the reference | Moderately worse than the reference | About the same as the reference | Moderately better than the reference | Significantly better than the reference |
| --- | --- | --- | --- | --- | --- |

|  | 0 | 25 | 50 | 75 | 100 |
| --- | --- | --- | --- | --- | --- |

| Play Sound Reference () | 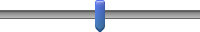 |
| --- | --- |
| Play Sound Anchor () | 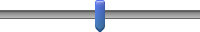 |
| Play Sound V+9 () | 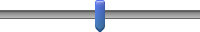 |
| Play Sound V-9 () | 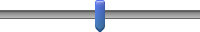 |

| Page Break |  |
| --- | --- |

| 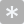 | 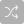 | 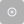 |
| --- | --- | --- |

Q69 Play Reference
A-EQ

|  | Significantly worse than the reference | Moderately worse than the reference | About the same as the reference | Moderately better than the reference | Significantly better than the reference |
| --- | --- | --- | --- | --- | --- |

|  | 0 | 25 | 50 | 75 | 100 |
| --- | --- | --- | --- | --- | --- |

| Play Sound Reference () | 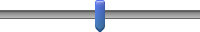 |
| --- | --- |
| Play Sound Anchor () | 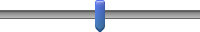 |
| Play Sound H+9 () | 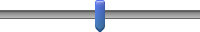 |
| Play Sound H-9 () | 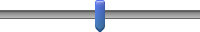 |
| Play Sound L+9 () | 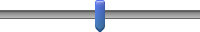 |
| Play Sound L-9 () | 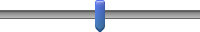 |
| Play Sound M+9 () | 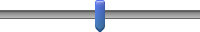 |
| Play Sound M-9 () | 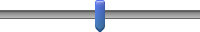 |

End of Block: A

Start of Block: B

| 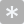 | 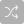 | 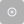 |
| --- | --- | --- |

Q70 Play Reference
B-VB

|  | Significantly worse than the reference | Moderately worse than the reference | About the same as the reference | Moderately better than the reference | Significantly better than the reference |
| --- | --- | --- | --- | --- | --- |

|  | 0 | 25 | 50 | 75 | 100 |
| --- | --- | --- | --- | --- | --- |

| Play Sound Reference () | 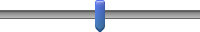 |
| --- | --- |
| Play Sound Anchor () | 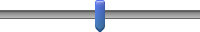 |
| Play Sound V+9 () | 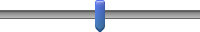 |
| Play Sound V-9 () | 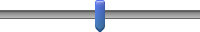 |

| Page Break |  |
| --- | --- |

| 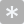 | 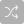 | 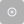 |
| --- | --- | --- |

Q71 Play Reference
B-EQ

|  | Significantly worse than the reference | Moderately worse than the reference | About the same as the reference | Moderately better than the reference | Significantly better than the reference |
| --- | --- | --- | --- | --- | --- |

|  | 0 | 25 | 50 | 75 | 100 |
| --- | --- | --- | --- | --- | --- |

| Play Sound Reference () | 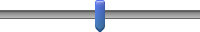 |
| --- | --- |
| Play Sound Anchor () | 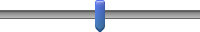 |
| Play Sound H+9 () | 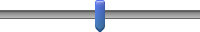 |
| Play Sound H-9 () | 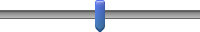 |
| Play Sound L+9 () | 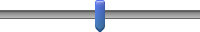 |
| Play Sound L-9 () | 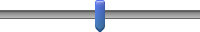 |
| Play Sound M+9 () | 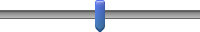 |
| Play Sound M-9 () | 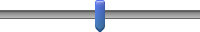 |

End of Block: B

Start of Block: C

| 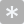 | 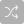 | 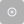 |
| --- | --- | --- |

Q72 Play Reference
C-VB

|  | Significantly worse than the reference | Moderately worse than the reference | About the same as the reference | Moderately better than the reference | Significantly better than the reference |
| --- | --- | --- | --- | --- | --- |

|  | 0 | 25 | 50 | 75 | 100 |
| --- | --- | --- | --- | --- | --- |

| Play Sound Reference () | 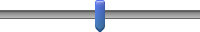 |
| --- | --- |
| Play Sound Anchor () | 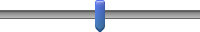 |
| Play Sound V+9 () | 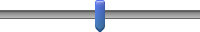 |
| Play Sound V-9 () | 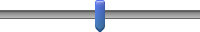 |

| Page Break |  |
| --- | --- |

| 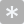 | 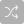 | 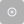 |
| --- | --- | --- |

Q73 Play Reference
C-EQ

|  | Significantly worse than the reference | Moderately worse than the reference | About the same as the reference | Moderately better than the reference | Significantly better than the reference |
| --- | --- | --- | --- | --- | --- |

|  | 0 | 25 | 50 | 75 | 100 |
| --- | --- | --- | --- | --- | --- |

| Play Sound Reference () | 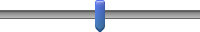 |
| --- | --- |
| Play Sound Anchor () | 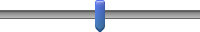 |
| Play Sound H+9 () | 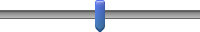 |
| Play Sound H-9 () | 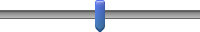 |
| Play Sound L+9 () | 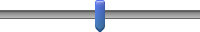 |
| Play Sound L-9 () | 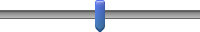 |
| Play Sound M+9 () | 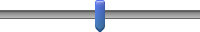 |
| Play Sound M-9 () | 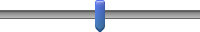 |

End of Block: C

Start of Block: D

| 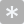 | 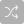 | 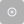 |
| --- | --- | --- |

Q74 Play Reference
D-VB

|  | Significantly worse than the reference | Moderately worse than the reference | About the same as the reference | Moderately better than the reference | Significantly better than the reference |
| --- | --- | --- | --- | --- | --- |

|  | 0 | 25 | 50 | 75 | 100 |
| --- | --- | --- | --- | --- | --- |

| Play Sound Reference () | 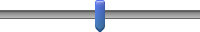 |
| --- | --- |
| Play Sound Anchor () | 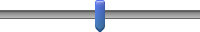 |
| Play Sound V+9 () | 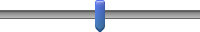 |
| Play Sound V-9 () | 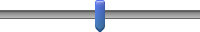 |

| Page Break |  |
| --- | --- |

| 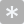 | 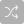 | 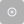 |
| --- | --- | --- |

Q75 Play Reference
D-EQ

|  | Significantly worse than the reference | Moderately worse than the reference | About the same as the reference | Moderately better than the reference | Significantly better than the reference |
| --- | --- | --- | --- | --- | --- |

|  | 0 | 25 | 50 | 75 | 100 |
| --- | --- | --- | --- | --- | --- |

| Play Sound Reference () | 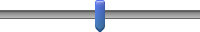 |
| --- | --- |
| Play Sound Anchor () | 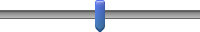 |
| Play Sound H+9 () | 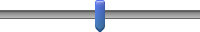 |
| Play Sound H-9 () | 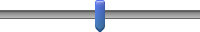 |
| Play Sound L+9 () | 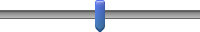 |
| Play Sound L-9 () | 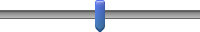 |
| Play Sound M+9 () | 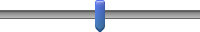 |
| Play Sound M-9 () | 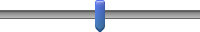 |

End of Block: D

Start of Block: E

| 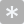 | 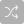 | 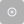 |
| --- | --- | --- |

Q76 Play Reference
E-VB

|  | Significantly worse than the reference | Moderately worse than the reference | About the same as the reference | Moderately better than the reference | Significantly better than the reference |
| --- | --- | --- | --- | --- | --- |

|  | 0 | 25 | 50 | 75 | 100 |
| --- | --- | --- | --- | --- | --- |

| Play Sound Reference () | 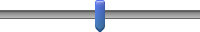 |
| --- | --- |
| Play Sound Anchor () | 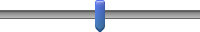 |
| Play Sound V+9 () | 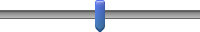 |
| Play Sound V-9 () | 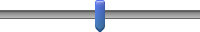 |

| Page Break |  |
| --- | --- |

| 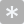 | 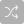 | 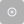 |
| --- | --- | --- |

Q77 Play Reference
E-EQ

|  | Significantly worse than the reference | Moderately worse than the reference | About the same as the reference | Moderately better than the reference | Significantly better than the reference |
| --- | --- | --- | --- | --- | --- |

|  | 0 | 25 | 50 | 75 | 100 |
| --- | --- | --- | --- | --- | --- |

| Play Sound Reference () | 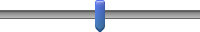 |
| --- | --- |
| Play Sound Anchor () | 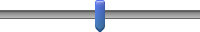 |
| Play Sound H+9 () | 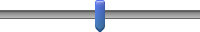 |
| Play Sound H-9 () | 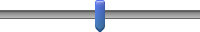 |
| Play Sound L+9 () | 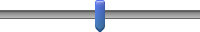 |
| Play Sound L-9 () | 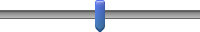 |
| Play Sound M+9 () | 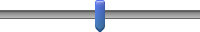 |
| Play Sound M-9 () | 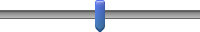 |

End of Block: E

Start of Block: F

| 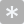 | 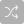 | 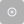 |
| --- | --- | --- |

Q78 Play Reference
F-VB

|  | Significantly worse than the reference | Moderately worse than the reference | About the same as the reference | Moderately better than the reference | Significantly better than the reference |
| --- | --- | --- | --- | --- | --- |

|  | 0 | 25 | 50 | 75 | 100 |
| --- | --- | --- | --- | --- | --- |

| Play Sound Reference () | 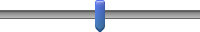 |
| --- | --- |
| Play Sound Anchor () | 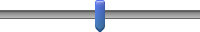 |
| Play Sound V+9 () | 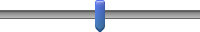 |
| Play Sound V-9 () | 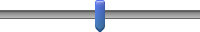 |

| Page Break |  |
| --- | --- |

| 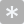 |  |  |
| --- | --- | --- |

Q79 Play Reference
F-EQ

|  | Significantly worse than the reference | Moderately worse than the reference | About the same as the reference | Moderately better than the reference | Significantly better than the reference |
| --- | --- | --- | --- | --- | --- |

|  | 0 | 25 | 50 | 75 | 100 |
| --- | --- | --- | --- | --- | --- |

| Play Sound Reference () |  |
| --- | --- |
| Play Sound Anchor () |  |
| Play Sound H+9 () |  |
| Play Sound H-9 () |  |
| Play Sound L+9 () |  |
| Play Sound L-9 () |  |
| Play Sound M+9 () |  |
| Play Sound M-9 () |  |

End of Block: F

Start of Block: G

|  |  |  |
| --- | --- | --- |

Q80 Play Reference
G-VB

|  | Significantly worse than the reference | Moderately worse than the reference | About the same as the reference | Moderately better than the reference | Significantly better than the reference |
| --- | --- | --- | --- | --- | --- |

|  | 0 | 25 | 50 | 75 | 100 |
| --- | --- | --- | --- | --- | --- |

| Play Sound Reference () |  |
| --- | --- |
| Play Sound Anchor () |  |
| Play Sound V+9 () |  |
| Play Sound V-9 () |  |

| Page Break |  |
| --- | --- |

|  |  |  |
| --- | --- | --- |

Q81 Play Reference
G-EQ

|  | Significantly worse than the reference | Moderately worse than the reference | About the same as the reference | Moderately better than the reference | Significantly better than the reference |
| --- | --- | --- | --- | --- | --- |

|  | 0 | 25 | 50 | 75 | 100 |
| --- | --- | --- | --- | --- | --- |

| Play Sound Reference () |  |
| --- | --- |
| Play Sound Anchor () |  |
| Play Sound H+9 () |  |
| Play Sound H-9 () |  |
| Play Sound L+9 () |  |
| Play Sound L-9 () |  |
| Play Sound M+9 () |  |
| Play Sound M-9 () |  |

End of Block: G

Start of Block: H

|  |  |  |
| --- | --- | --- |

Q82 Play Reference
H-VB

|  | Significantly worse than the reference | Moderately worse than the reference | About the same as the reference | Moderately better than the reference | Significantly better than the reference |
| --- | --- | --- | --- | --- | --- |

|  | 0 | 25 | 50 | 75 | 100 |
| --- | --- | --- | --- | --- | --- |

| Play Sound Reference () |  |
| --- | --- |
| Play Sound Anchor () |  |
| Play Sound V+9 () |  |
| Play Sound V-9 () |  |

| Page Break |  |
| --- | --- |

|  |  |  |
| --- | --- | --- |

Q83 Play Reference
H-EQ

|  | Significantly worse than the reference | Moderately worse than the reference | About the same as the reference | Moderately better than the reference | Significantly better than the reference |
| --- | --- | --- | --- | --- | --- |

|  | 0 | 25 | 50 | 75 | 100 |
| --- | --- | --- | --- | --- | --- |

| Play Sound Reference () |  |
| --- | --- |
| Play Sound Anchor () |  |
| Play Sound H+9 () |  |
| Play Sound H-9 () |  |
| Play Sound L+9 () |  |
| Play Sound L-9 () |  |
| Play Sound M+9 () |  |
| Play Sound M-9 () |  |

End of Block: H

Start of Block: I

|  |  |  |
| --- | --- | --- |

Q84 Play Reference
I-VB

|  | Significantly worse than the reference | Moderately worse than the reference | About the same as the reference | Moderately better than the reference | Significantly better than the reference |
| --- | --- | --- | --- | --- | --- |

|  | 0 | 25 | 50 | 75 | 100 |
| --- | --- | --- | --- | --- | --- |

| Play Sound Reference () |  |
| --- | --- |
| Play Sound Anchor () |  |
| Play Sound V+9 () |  |
| Play Sound V-9 () |  |

| Page Break |  |
| --- | --- |

|  |  |  |
| --- | --- | --- |

Q85 Play Reference
I-EQ

|  | Significantly worse than the reference | Moderately worse than the reference | About the same as the reference | Moderately better than the reference | Significantly better than the reference |
| --- | --- | --- | --- | --- | --- |

|  | 0 | 25 | 50 | 75 | 100 |
| --- | --- | --- | --- | --- | --- |

| Play Sound Reference () |  |
| --- | --- |
| Play Sound Anchor () |  |
| Play Sound H+9 () |  |
| Play Sound H-9 () |  |
| Play Sound L+9 () |  |
| Play Sound L-9 () |  |
| Play Sound M+9 () |  |
| Play Sound M-9 () |  |

End of Block: I

Start of Block: J

|  |  |  |
| --- | --- | --- |

Q86 Play Reference
J-VB

|  | Significantly worse than the reference | Moderately worse than the reference | About the same as the reference | Moderately better than the reference | Significantly better than the reference |
| --- | --- | --- | --- | --- | --- |

|  | 0 | 25 | 50 | 75 | 100 |
| --- | --- | --- | --- | --- | --- |

| Play Sound Reference () |  |
| --- | --- |
| Play Sound Anchor () |  |
| Play Sound V+9 () |  |
| Play Sound V-9 () |  |

| Page Break |  |
| --- | --- |

|  |  |  |
| --- | --- | --- |

Q87 Play Reference
J-EQ

|  | Significantly worse than the reference | Moderately worse than the reference | About the same as the reference | Moderately better than the reference | Significantly better than the reference |
| --- | --- | --- | --- | --- | --- |

|  | 0 | 25 | 50 | 75 | 100 |
| --- | --- | --- | --- | --- | --- |

| Play Sound Reference () |  |
| --- | --- |
| Play Sound Anchor () |  |
| Play Sound H+9 () |  |
| Play Sound H-9 () |  |
| Play Sound L+9 () |  |
| Play Sound L-9 () |  |
| Play Sound M+9 () |  |
| Play Sound M-9 () |  |

End of Block: J

Start of Block: Thank you!

Q88 We thank you for your interest and participation in this study!

End of Block: Thank you!
